# *DICER1*-related tumour predisposition mutations increase 3p-miRNA function and HERVH activity

**DOI:** 10.1038/s41467-026-76094-2

**Published:** 2026-07-25

**Authors:** Katrina Gordon, Nicolas Bellora, Jeroen Witteveldt, Joanna K. Wojtus, Felix Mueller, Katharina J. Kases, Pilar G. Marchante, Guillermo Peris, Shelagh Boyle, Sara R. Heras, Atlanta G. Cook, Cei Abreu-Goodger, Sara Macias

**Affiliations:** 1 Institute of Immunology and Infection Research, Ashworth Laboratories, School of Biological Sciences, University of Edinburgh, Edinburgh, UK; 2 National Scientific and Technical Research Council (CONICET), San Carlos de Bariloche, Argentina; 3 GENYO, Centre for Genomics and Oncological Research: Pfizer/University of Granada/Andalusian Regional Government, PTS Granada, Granada, Spain; 4 Dept. Biochemistry and Molecular Biology II, Faculty of Pharmacy, University of Granada, Campus Universitario de Cartuja, Granada, Spain; 5 Department of Computer Languages and Systems, Universitat Jaume I, Castellon de la Plana, Spain; 6 MRC Human Genetics Unit, Institute of Genetics and Cancer, University of Edinburgh, Edinburgh, UK; 7 Institute of Quantitative Biology, Biochemistry and Biotechnology, Max Born Crescent, University of Edinburgh, Edinburgh, UK; 8 Institute of Ecology and Evolution, School of Biological Sciences, The University of Edinburgh, Edinburgh, UK

**Keywords:** Small RNAs, Cancer epigenetics, Transposition

## Abstract

The *DICER1* gene is mutated in cancer, including in *DICER1*-related tumour predisposition. Cancer-associated hotspot mutations have been reported in both catalytic domains of DICER and are predicted to disrupt miRNA biogenesis. To understand how these hotspot mutations contribute to cancer development, we generate cell lines harbouring single amino acid substitutions within the catalytic RNase IIIa (S1344L) or RNase IIIb (D1709N) domains of the endogenous *DICER1* gene. Here we show that both mutations result in a widespread loss of 5p miRNAs, and an increase in 3p passenger strands loading into AGO2. The shared similarities between both mutants can be attributed to the structural proximity of the S1344 residue to the RNase IIIb catalytic centre. Functionally, we find that changes in the repertoire of miRNAs loaded into AGO2 result in altered gene expression, impacting critical pathways for cancer development. Additionally, our results indicate that inactivating the processing activity of DICER does not result in genomic instability. Instead, mutations cause upregulation of transposable elements, including the human endogenous retrovirus H through miRNA-independent mechanisms. This suggests that both canonical and non-canonical DICER functions are important to understand *DICER1*-related tumour predisposition.

## Abstract (web summary)

DICER1 is often mutated in cancer, including in DICER1-related tumour predisposition. Here, the authors show that cancer-associated DICER1 mutations shift microRNA processing and reactivate HERVH endogenous retroviruses.

## Introduction

DICER is an essential enzyme for the production of microRNAs (miRNAs). In humans, the canonical miRNA biogenesis pathway consists of two processing steps. The first step is nuclear and is carried out by the Microprocessor complex, composed of DROSHA and DGCR8, which generates precursor miRNAs (pre-miRNA). In the second cytoplasmic processing step, DICER cleaves the pre-miRNA hairpin to remove the loop. This generates a miRNA duplex containing both 5p and 3p miRNAs. Usually, one of the miRNA strands is preferentially loaded into an Argonaute protein (AGO), named the “guide” strand. The other strand is the “passenger” and it is usually degraded. This selection seems to be dictated by the thermodynamic stability of the miRNA duplex ends and the 5′ nucleotide identity (reviewed in ref. ^1^). In vitro, AGO displays preference for 5′ uracil and loads the strand with the less stable 5′ end^2^. However, a significant proportion of miRNAs do not follow these rules, and strand selection seems to be context or cell-type-dependent^3^. The seed sequence, in positions 2 to 8 relative to the 5′ end of the miRNA, is critical for directing the RNA-induced silencing complex (RISC), containing AGO loaded with miRNAs, by complementary sites in the 3′ untranslated region (UTR) of target mRNAs.

While insects, nematodes and plants possess duplicated *DICER* genes, with specialised paralogs for miRNA biogenesis or the production of small interfering RNAs (siRNAs), mammals only have a single *DICER1* gene that is required to process both miRNA and siRNA precursors (reviewed in ref. ^4^). Although it is still unclear if mammals possess a functional endogenous siRNA (endo-siRNA) pathway, miRNA-independent functions of mammalian DICER have been reported. These include the maintenance of genomic stability and chromosome segregation, as well as preventing the accumulation of transcripts derived from the major satellite repeats from pericentromeric regions^5–11^. In addition, DICER has also been implicated in regulating other types of repeats, including retrotransposons (reviewed in refs. ^12,13^). In support of this, mouse Dicer was found to repress the expression of LTR and LINE-1 retrotransposons during early development and embryonic stem cells (mESCs)^14–17^. Although the mechanisms driving transposon silencing remain unclear, LINE-1 transcripts are predicted to generate siRNAs with silencing activity. The LINE-1 5′UTR contains a bidirectional promoter, presumably resulting in the formation of dsRNA that can act as a substrate for DICER cleavage^18^. Other endo-siRNAs have been detected in mouse oocytes and mESCs. These are predicted to regulate the main retrotransposon families, LINE, SINE and LTR^19–21^.

*DICER1* mutations have been identified in *DICER1*-related tumour predisposition (DRTP). This disease increases the predisposition to both benign and malignant tumours. Patients typically carry an inherited loss-of-function mutation in one *DICER1* allele and acquire a second somatic mutation later in life. While the loss-of-function germline mutations can occur in any region of the protein, the somatic mutations usually occur within the catalytically active, metal-ion binding residues^22^. These include amino acid substitutions in both RNase IIIa and IIIb endonucleolytic domains. The RNase IIIa domain cleaves the 3p-arm within the pre-miRNA hairpin, while the RNase IIIb cleaves the 5p-miRNA (reviewed in ref. ^23^). Despite being in different RNase III domains, most cancer-associated mutations are predicted to only affect 5p miRNA production. Intriguingly, there does not seem to be a selective advantage for mutations affecting 3p miRNA production in cancer^24^.

Considering that DICER is an essential protein for cell survival, it is unclear how inactivating its catalytical activity can contribute to cancer^25,26^. To investigate this, we have generated human cells recapitulating cancer-associated hotspot mutations using CRISPR/Cas9 editing. By comparing different mutations in the RNase IIIa and IIIb domains, we have confirmed that mutations in either domain results in inactivation of 5p miRNA production. However, there is a concomitant increased loading of 3p miRNAs into AGO2, leading to numerous cases of miRNA arm switching. This results in defective silencing of 5p-miRNA targets and increased repression of 3p-target mRNAs. Our results show that the hotspot mutation S1344L in the RNase IIIa also affects 5p-miRNA cleavage, but to a lesser extent than RNase IIIb D1709N. Analysis of the DICER protein structure showed that S1344 is spatially in proximity to the RNase IIIb active site, supporting its role in 5p-miRNA processing. Interestingly, despite having a phenotype of defective growth, *DICER1*-mutant cell lines showed increased cell migration, providing an explanation of how these mutations can predispose to cancer progression. Mechanistically, we show that these DRTP mutations do not cause genomic instability or chromosome abnormalities. Instead, these mutations are associated with upregulation of endogenous retroviruses (ERVs) typical of a “pluripotency” profile. Crucially, we observed a similar miRNA and TE dysregulated phenotype in *DICER1*-mutated tumours. All this together suggest that DRTP mutations not only cause a loss of 5p-miRNA, but also an increase in 3p-miRNA function and TE expression.

## Results

### Generation of cell lines containing DRTP-associated mutations

**Fig. 1:**
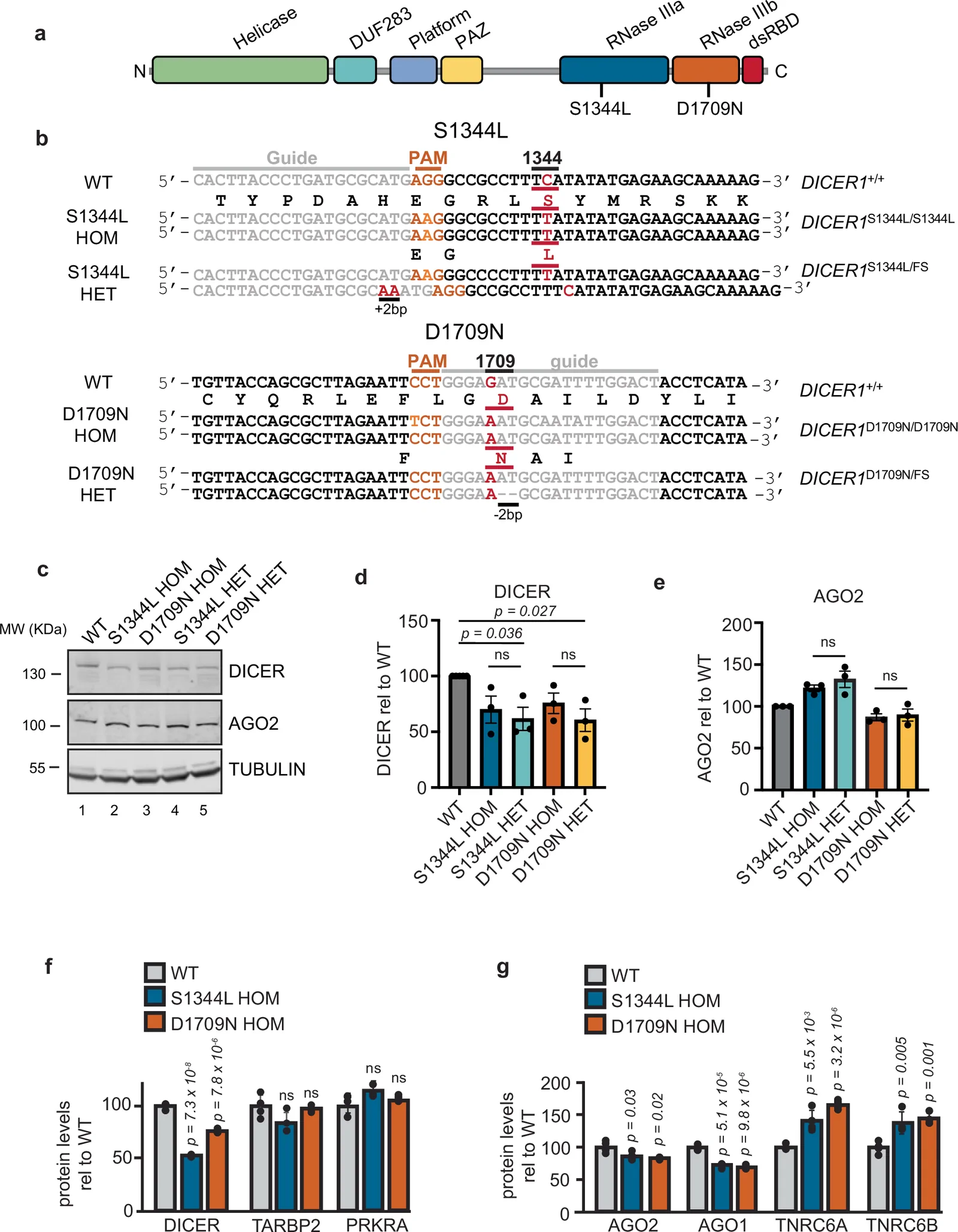
Generation of *DICER1*-mutant PA-1 cell lines harbouring point mutations in either RNase IIIa or RNase IIIb domain. **a** Schematic of DICER protein showing functional domains as indicates (DUF: domain of unknown function). Targeted point mutations at amino acid residue S1344L and D1709N in the RNase IIIa and RNase IIIb domains are shown. **b** CRISPR/Cas9 knock-in strategy showing the sequence of the targeted region in PA-1 wild type (WT) cells and the corresponding sequence changes in each of the mutant clones as indicated with amino acid change S1344L and D1709N, Guides (grey) and PAM (orange). Both alleles are shown for each of the mutants, homozygous (S1344L HOM and D1709N HOM) and frameshift (FS) allele are indicated in the compound heterozygous lines (S1344L HET and D1709N HET). Genotypes are as follows: S1344L HOM (*DICER1*^S1344L/S1344L^), S1344L HET (*DICER1*^S1344L/FS^), D1709N HOM (*DICER1*^D1709N/D1709N^) and D1709N-HET (*DICER1*^D1709NL/FS^). **c** Western blot analysis of DICER and AGO2 protein levels in WT, S1344L and D1709N mutant cell lines, using TUBULIN as a loading control (**d** and **e**) Quantification of DICER (**d**) and AGO2 (**e**) protein levels relative to WT. Error bars indicate mean values ± S.D. from three independent experiments with individual data points shown. Molecular weights (MW) in KDa are indicated (**f** and **g**) Proteomic data analysis of DICER-binding partners TARBP2 and PRKRA (**f**) and components of the miRISC complex, AGO1/2 and TNRC6A/B (**g**) in WT and HOM mutants from total cell extracts. Data are presented as the average ± S.D. from four independent experiments. For all panels, error bars indicate mean values ± SD followed by one-way ANOVA and Dunnett’s multiple comparison test (for **d** and **e**), or Welch’s *t*-test (for **f** and **g**). Non-significant comparisons are shown as (ns), significance is indicated with exact *p* values.

To study the role of *DICER1*-related tumour predisposition (DRTP) associated hotspot mutations, we generated human cell lines containing single-amino acid substitutions in *DICER1* using CRISPR/Cas9. We used PA-1 cells; a diploid human embryonic cell line derived from an ovarian teratocarcinoma. We introduced the S1344L substitution in the RNase IIIa domain and the D1709N substitution at the RNase IIIb domain, which are typical hotspot mutations found in DRTP patients (Fig. 1a). Cell lines where both alleles were modified with the same mutations (S1344L or D1709N) were named homozygote mutants (HOM). We also generated compound heterozygote (HET) mutants. HET cell lines contained only one allele mutated either as S1344L or D1709N, while the second allele contained an *indel* mutation generating a premature-termination codon (PTC), driving the resulting mRNA to be degraded by nonsense-mediated decay (NMD) (Fig. 1b). The HET cell lines are representative of the genetic composition observed in DRTP patients, where one allele contains a loss-of-function mutation that results in loss of protein production, while the second allele acquires a hotspot mutation in RNase IIIa or IIIb sites. No knock-out cell lines were obtained for the *DICER1* gene, suggesting that in human embryonic teratocarcinoma cells, DICER is essential for viability as observed in human embryonic stem cells^26^.

We first assessed DICER and AGO2 protein levels by western blotting (Fig. 1c). No significant differences were observed between HET and HOM mutants, despite HETs only producing DICER protein from a single allele (Fig. 1d, e). In agreement, *DICER1*^+/−^ PA-1 cells, where only a single allele is inactivated, did not display significant changes in miRNA expression, arguing against *DICER1* haploinsufficiency (Supplementary Fig. 1). Proteomics data confirmed that *DICER1* mutations did not result in significant changes in the protein levels of its co-factors, TARBP2 or PRKRA (also known as PACT) (Fig. 1f), while AGO2 and AGO1 displayed significantly decreased levels (Fig. 1g). Other components of the RNA-inducing silencing complex, such as TNRC6A and B, were not decreased by *DICER1* mutations (Fig. 1g and Supplementary Data 1).

### Increased cell migration in DRTP mutant cells

**Fig. 2:**
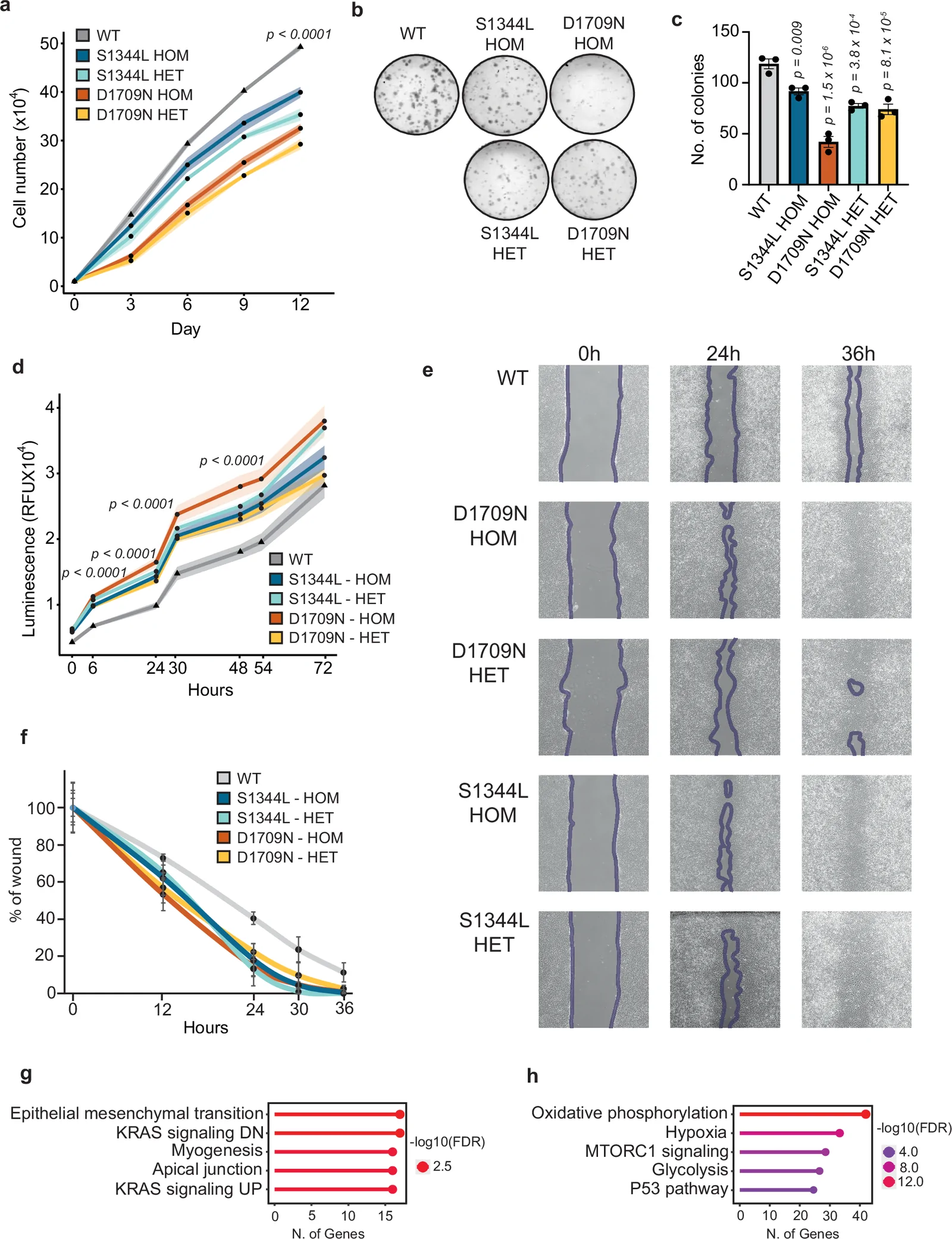
Differences in cellular phenotypes in the *DICER1*-mutant cell lines. **a** Proliferation rates of the four *DICER1* mutant cell lines were compared to WT PA-1 cells. Shaded lines indicate mean values ± SD of three independent replicates. All *DICER1*-mutant cells are significantly different from WT, as indicated by *p* adj < 0.0001 by one-way ANOVA followed by Šídák’s multiple comparison test. **b** Representative images of crystal violet-stained colonies indicating the colony formation capacity of each of the cell lines. **c** Quantification of the number of colonies. Error bars indicate mean values ± SD of three independent replicates compared to WT. One-way ANOVA followed by Tukey’s multiple comparison test was used to calculate *p*-val. **d** Metabolic activity across the different cell lines was assessed by quantifying cellular ATP via a luciferase reaction and measuring the resulting luminescence (RFU, relative fluorescence units) over time. Shaded lines indicate ± SD of four independent replicates. All *DICER1*-mutant cells are significantly different from WT at the indicated time points, *p* adj <0.0001 by one-way ANOVA followed by Šídák’s multiple comparison test. **e** Representative phase contrast images of wound healing assays at times 0, 24 and 36 h after initial wound for all cell lines. **f** Quantitative analysis of wound closure expressed as a percentage of the original wound. Data are presented as the average ± SD from three independent experiments (*n* = 3). All mutants (except S1344L-HET at *t* = 12 h) have significantly higher rates of wound closure at all time points compared to WT (*p* < 0.007). Significance was calculated by one-way ANOVA, followed by a Tukey HSD/Tukey Kramer post-hoc test. **g**, **h** Hallmark gene sets for the commonly differentially expressed up (**g**) and down (**h**) regulated genes in DICER mutants (S1344L HOM and D1709N HOM/HET) compared to WT PA-1 cells (only the top 5 gene sets are shown). *P*-val was calculated using a one-sided the hypergeometric test, and adjusted for multiple comparisons using the Benjamini–Hochberg False Discovery Rate (FDR) method: value is shown as −log10 (FDR).

Considering that *DICER1* is an essential gene in both mouse and in human cells, it is unclear how its mutation contributes to tumorigenesis. To explore the mechanisms by which DICER mutation predisposes to cancer, we decided to investigate different phenotypes of the generated cell lines. In agreement with its reported role in cell survival and cell cycle progression, we confirmed that all *DICER1* mutant cells showed significantly lower replication rates compared to wild-type (WT) cells (Fig. 2a and Supplementary Fig. 2A). These mutations also resulted in a decreased capacity for colony formation (Fig. 2b, c and Supplementary Fig. 2B). On the other hand, mutant *DICER1* cell lines display increased metabolic activity, as determined by a luminescence-based assay for intracellular ATP levels (Fig. 2d). The mutants also displayed a significantly increased ability to migrate in a wound healing assay (Fig. 2e, f and Supplementary Fig. 2C). The migration capacity of *DICER1*-mutant cell lines suggest that these mutations could increase the metastatic potential.

To investigate if the *DICER1-*mutant cell lines displayed changes in gene expression supporting these phenotypes, we performed differential gene expression analyses of total RNA high-throughput sequencing in both S1344L and D1709N cell lines compared to WT. D1709N HET and HOM cells were combined in the differential expression analysis as they behaved similarly. Although D1709N cells showed more significantly expressed genes than S1344L, there was a clear overlap between the two mutants (Supplementary Data 2). More than 80% of the genes that were differentially expressed in the S1334L mutant cell line were also differentially expressed in D1709N cells. Considering that both mutations resulted in similar cellular phenotypes, we decided to focus our analyses on the commonly dysregulated genes. Genes upregulated upon *DICER1* mutations (*n* = 1306) were associated with “epithelial to mesenchymal transition (EMT)” and “KRAS signalling” (Fig. 2g). We confirmed that critical proteins within the EMT pathway were upregulated in mutant cells at the protein level (Supplementary Fig. 3). These results were in agreement with the increased migration observed by *DICER1* mutant cells in the wound healing assay, as EMT is associated with tissue repair, but also increased motility, invasiveness, metastasis and enhanced resistance to apoptosis^27,28^. KRAS signalling is also associated with increased invasion and metastasis, and constitutive activation of KRAS is observed in tumours^29^. On the other hand, commonly downregulated genes (*n* = 1174) were associated with “oxidative phosphorylation” and “MTORC1 signalling” (Fig. 2h). These pathways could be potentially relevant to the defects we observed in cell growth and alterations in the metabolic state of *DICER1* mutant cells. All these findings suggest that despite the critical role for DICER in cell survival, *DICER1*-related tumour predisposition mutations cause increased cell migration, thus explaining the role of this gene in cancer progression.

### S1344L and D1709N result both in 5p-miRNA loss and 3p-miRNA gain

**Fig. 3:**
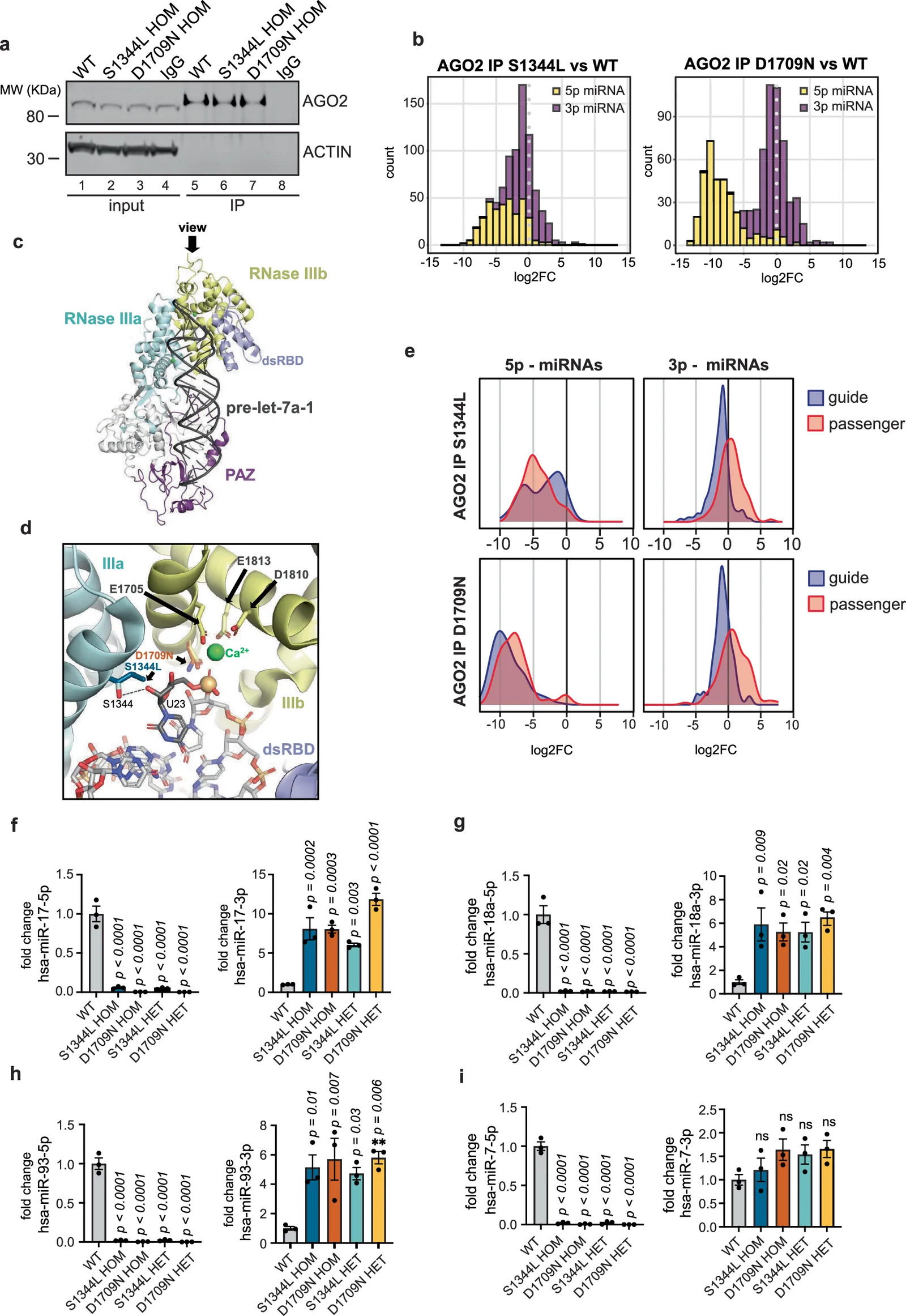
Accumulation of 3p-miRNAs in *DICER1*-mutated cell lines. **a** AGO2 immunoprecipitation (IP) levels in WT, S1344L HOM and D1709N HOM cell lines (lanes 5–7) by AGO2 western blot. IgG was used as a negative control (lane 8). Input levels are shown in lanes 1–4. Western blot against ACTIN was used a negative control for the IP. Molecular weight (MW) markers are included in KDa. Data is one representative experiment out of 3 independent experiments with similar results. Additional experiments are provided in the Source Data file. **b** MiRNA counts (*y*-axis) showing the log2FC distribution of 5p and 3p miRNAs in the S1344L HOM (left) and D1709N HOM (right) mutants relative to WT using the AGO2-IP dataset. **c** Overview of pre-let-7a-1 bound to human DICER in the dicing state (PDBid 7XW2). PAZ (violet), RNase IIIa (cyan), RNase IIIb (yellow) and dsRNA binding domain (dsRBD, light blue) are highlighted. **d** A viewpoint taken from the top of the pre-miR helix, showing the active site of RNase IIIb. A divalent cation (Ca^2+^, green) in the active site is coordinated by four acidic side chain (including E1705, D1810 and D1813) with D1709 nearby. Ser1344 interacts (black dotted line) with the ribose 2′OH of U23. The phosphate group of U23, which is the site of hydrolysis, is highlighted with an orange sphere at the phosphate atom. Superimposed is the leucine sidechain (teal) of a S1344L mutant and the asparagine sidechain (orange) of D1709N. **e** Density plots showing the change in distribution of miRNAs (divided into 5p/3p and guide/passenger) in the mutants relative to WT using the AGO2-IP dataset. Guide strand (blue) and passenger strand (red). For guide definition, see Supplementary Fig. 4C. **f–i** RT-qPCR analysis for miRNA levels in mutant cell lines relative to WT. Both 5p (left) and 3p (right) miRNA abundance was measured for miR-17, miR-18a, miR-93 and miR-7. SNORD44 was used as a normaliser. Error bars represent the average of 3 biological replicates ± SD. *P*-adj values are indicated, ns (not significant), by one-way ANOVA followed by Šídák’s multiple comparison test.

To understand the mechanism by which DRTP mutations caused these altered cellular phenotypes, we initially assessed their effects on miRNA production. We measured miRNA levels in cells using two alternative approaches, small RNA-sequencing and high-throughput RNA sequencing of AGO2 immunoprecipitates (IP) (Fig. 3a and Supplementary Data 3 and 4). By comparing these two datasets, we can quantify which miRNAs are differentially expressed and which miRNAs are differentially loaded into AGO2. Both small RNA and AGO2 IP libraries were enriched in 21–23 nt long RNAs, as expected (Supplementary Fig. 4A). This analysis revealed that despite being in two distinct RNase III domains, both S1344L and D1709N mutations resulted in decreased abundance of 5p-miRNAs in both the small RNA and AGO2 IP datasets, relative to WT cells (Fig. 3b, Supplementary Fig. 4B and Supplementary Data 3 and 4). This suggests that both mutations have a similar effect on DICER activity. As previously noted, the S1344 residue of DICER is structurally close to the catalytic site responsible for 5p-miRNA cleavage, despite being in the RNase IIIa domain (Fig. 3c, d)^24^. The importance of S1344 is yet more apparent on analysis of an experimentally determined structure of human DICER in its dicing state, where S1344 forms a hydrogen bond with the 2′OH of the 23rd base from the 5′ end, orienting the backbone phosphate group linking the 22nd and 23rd nucleotides close to the RNase IIIb catalytic divalent cation^30^. We modelled the S1344L mutation in silico using coordinates from the DICER pre-miRNA dicing state (7XW2)^30^. Substituting the serine with leucine in position 1344 generates a steric clash with the pre-mRNA backbone near the active site of the RNase IIIb domain and so may prevent correct positioning of the 23rd phosphate group in the active site (Fig. 3d). On the other hand, divalent cations in the active site are coordinated by four acidic side chain (E1705, D1709, D1810 and E1813) that are all known hotspot sites for *DICER1* mutations^24^. D1709 is reported to bind a Mg^2+^ ion in the DICER active site that was not observed in the dicing state^31^. By modelling the D1709N mutation, we noted that this neutralizes the negative charge at this residue position, which may interfere with the catalytic metal ion binding to the enzyme. The difference in structural consequences of mutating the D1709 vs S1344 position explains why D1709N displays a more pronounced 5p miRNA loss than S1344L.

Contrary to the marked decrease in 5p-miRNAs, mutations in DICER resulted in an increased in abundance for a subset of 3p-miRNAs, in both the small RNA and AGO2 IP dataset (Fig. 3b and Supplementary Fig. 4B). First, we found that highly abundant 3p miRNAs in WT cells, such as miR-302-3p, were not upregulated in comparison with other less abundant 3p miRNAs (Supplementary Data 3 and 4). We then investigated whether these differences were dictated by the initial abundance of the 3p miRNA, being an abundant guide or alternatively, a less abundant passenger. To test this possibility, we first classified all 5p and 3p miRNAs into guides or passengers depending on their abundance in WT cells (Supplementary Fig. 4C). MiRNAs with similar abundance for the 5p and 3p strands were excluded from this analysis. We next plotted the differences in expression of 3p guide and passenger miRNAs, and whilst 3p guides did not change expression or were slightly reduced in *DICER1* mutants, 3p passengers showed increased abundance in both the small RNA and the AGO2 IP datasets (Fig. 3e and Supplementary Figs. 4B and 5A). Both the S1344L and D1709N mutants behaved similarly, suggesting that this defect is not the result of increased processing of the 3p miRNA, but increased loading efficiency into AGO2, in cases where the 5p miRNA guide cannot be loaded. We conclude that *DICER1* mutations cause a general loss of 5p miRNAs in addition to a gain of 3p miRNAs that are  lowly expressed in healthy or WT cells. In some extreme cases, this results in a complete miRNA strand switching, from 5p to 3p, as shown for miR-17, miR-18a and miR-93 by RT-qPCR, but not for miR-7 (Fig. 3f–i and Supplementary Fig. 5B).

**Fig. 4:**
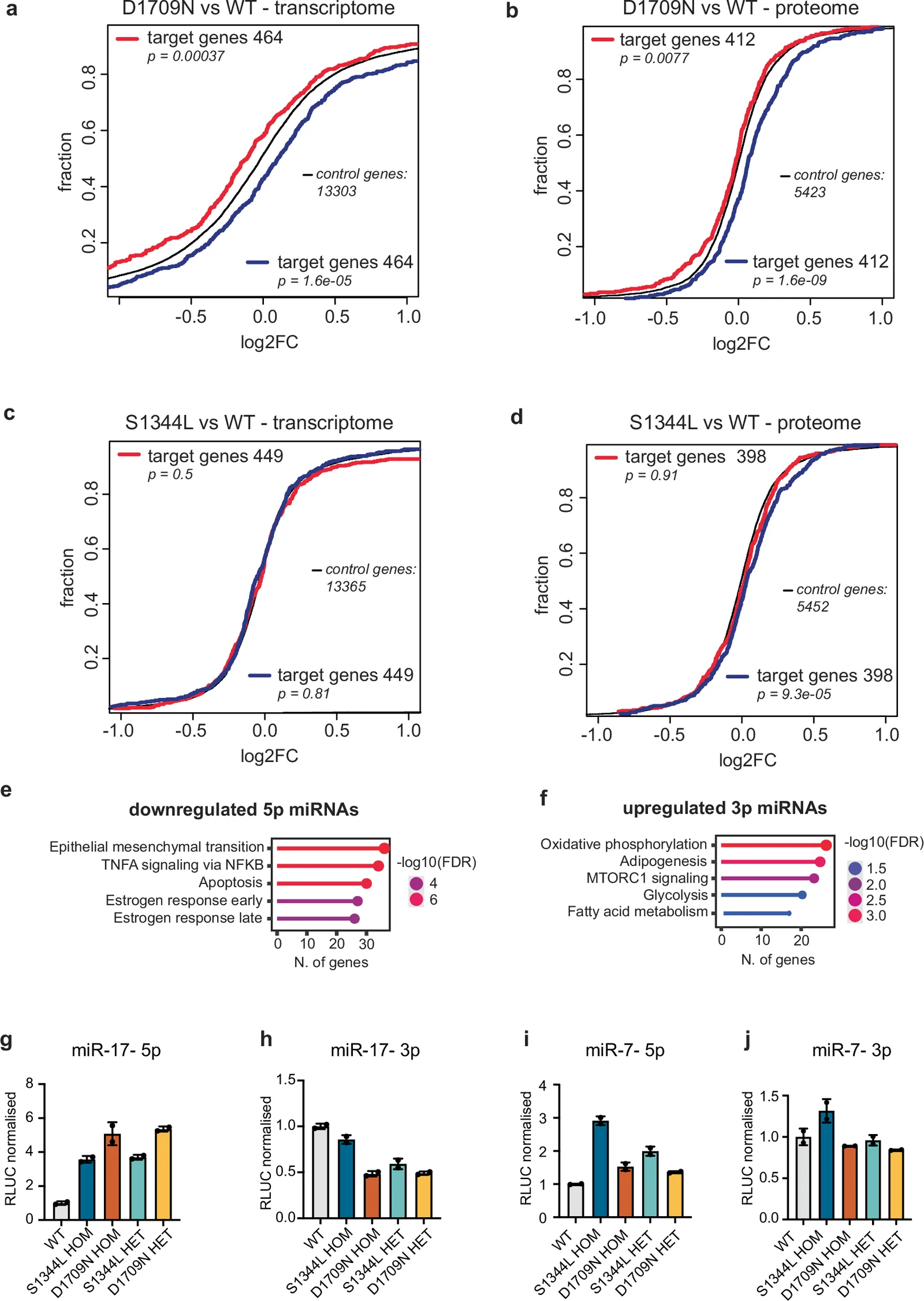
Impact of 3p and 5p-dysregulation in DICER1 mutated cell lines. **a** Cumulative distribution function (CDF) vs log2 fold-changes of predicted gene targets for upregulated (red) and downregulated (blue) top 10 miRNAs in the D1709N mutants (HOM/HET) compared to WT, measured by total RNA-seq (**a**) or total cell proteomics (**b**). **c** Cumulative distribution function (CDF) vs log2 fold-changes of predicted gene targets of upregulated (red) and downregulated (blue) top10 miRNAs in the S1344L HOM mutant compared to WT, measured by total RNA-seq (**c**) and total cell proteomics (**d**). The *p*-value indicates if log2 fold-changes (log2FC) of target genes are significantly down (red) or upregulated (blue) compared to control genes (Wilcoxon Rank Sum one-tailed test). **a–d** the 10 most highly expressed miRNAs were selected from the significantly up/downregulated in each mutant. The top 500 targets (according to TargetScan cumulative weighted context++ scores) for these 10 miRNAs were selected, and target genes that were also predicted for a miRNA regulated in the opposite direction were excluded (see “Methods”). Figure panels show the number of remaining genes. Black lines represent the log2FC distribution of all measured genes, after removal of the top 2000 predicted targets (1000 for each of up and downregulated miRNAs). **e** Hallmark gene sets of the predicted gene targets for the top 10 downregulated miRNAs (5p), which were significantly increased in the transcriptomic data (log fold 2 > 0.5, *p*-adj. <0.05), common to both mutants. **f** Hallmark gene sets of the predicted gene targets for the top 10 upregulated miRNAs (3p), which were significantly decreased in the transcriptomic data (log fold 2 > 0.5, *p*-adj. <0.05), common to both mutants. *P*-val for (**e**) and (**f**) were calculated using a one-sided hypergeometric test and adjusted for multiple comparisons using the Benjamini–Hochberg False Discovery Rate (FDR) method: values are shown as −log10 (FDR). (**g-j**) WT and *DICER1* mutant cell lines transfected with psiCHECK2 constructs containing complementary binding sites for 5p or 3p miRNA (miR-17 and miR-7) as part of the 3′UTR of the RLUC gene. Data are presented as normalised Relative Luminescence Units (RLUC) for two independent experiments.

The unexpected accumulation of 3p-miRNAs led us to investigate if this could be contributing to alterations in post-transcriptional gene silencing. To this end, we first analysed the cumulative distribution function (CDF) of genes predicted to be targeted by the top 10 dysregulated 3p and 5p-miRNAs. This analysis revealed that the top 500 predicted targets of the increased 3p miRNAs were destabilised in D1709N cells, both at the transcriptomic and proteomic level (Fig. 4a, b). On the other hand, targets of the 5p-miRNAs increased their steady state levels, as 5p-miRNAs were globally decreased in D1709N mutant cell lines, again at the transcriptomic and proteomic level (Fig. 4a, b). Using the same approach, we measured the effect of the S1344L mutation. In this case, no significant changes were observed, except for the 5p-miRNA targets at the proteome level (Fig. 4c, d), confirming that the molecular defects observed by S1344L mutation are less pronounced than D1709N mutants.

We also explored the pathways associated with the predicted targets for the differentially expressed 5p and 3p miRNAs. We focused on targets that were significantly dysregulated in the RNA-seq datasets of both mutants. Interestingly, the predicted targets of the downregulated 5p miRNAs were involved in epithelial to mesenchymal transition (EMT) (Fig. 4e). On the other hand, the predicted targets of the upregulated 3p miRNAs were associated with metabolic pathways (Fig. 4f). It seems that both 5p and 3p miRNA dysregulation can contribute to defective post-transcriptional gene silencing in *DICER1* mutant cells, and that these changes may contribute to the altered cellular behaviours observed in wound healing and metabolism.

To further confirm the functional relevance of 5p and 3p-miRNA dysregulation, we used luciferase-based sensors to quantify the silencing effect of these miRNAs (Fig. 4g–j). The RLUC (*Renilla* luciferase) reporter was fused to a 3′UTR containing complementary binding sites for miR-17-5p or -3p miRNAs. As a control, we included miR-7, as the 5p was depleted upon DICER mutation, while the 3p remained unchanged (Fig. 3h). As expected, the 5p miRNA sensors displayed increased RLUC levels in the DICER mutant cell lines, as both 5p miRNAs are depleted in mutant cells (Fig. 4g and i). The miR-17-3p sensor decreased its expression in DICER mutant cell lines, and this is consistent with the increased levels of miR-17-3p (Fig. 4h). The miR-7-3p sensor remained mostly unchanged, as expected, since no significant changes in miR-7-3p levels were observed (Fig. 4j). All these results suggest that both 5p and 3p dysregulation contribute to the alterations of the gene expression profile of DICER mutant cells.

### Dysregulated ERV profile in DRTP cell models

**Fig. 5:**
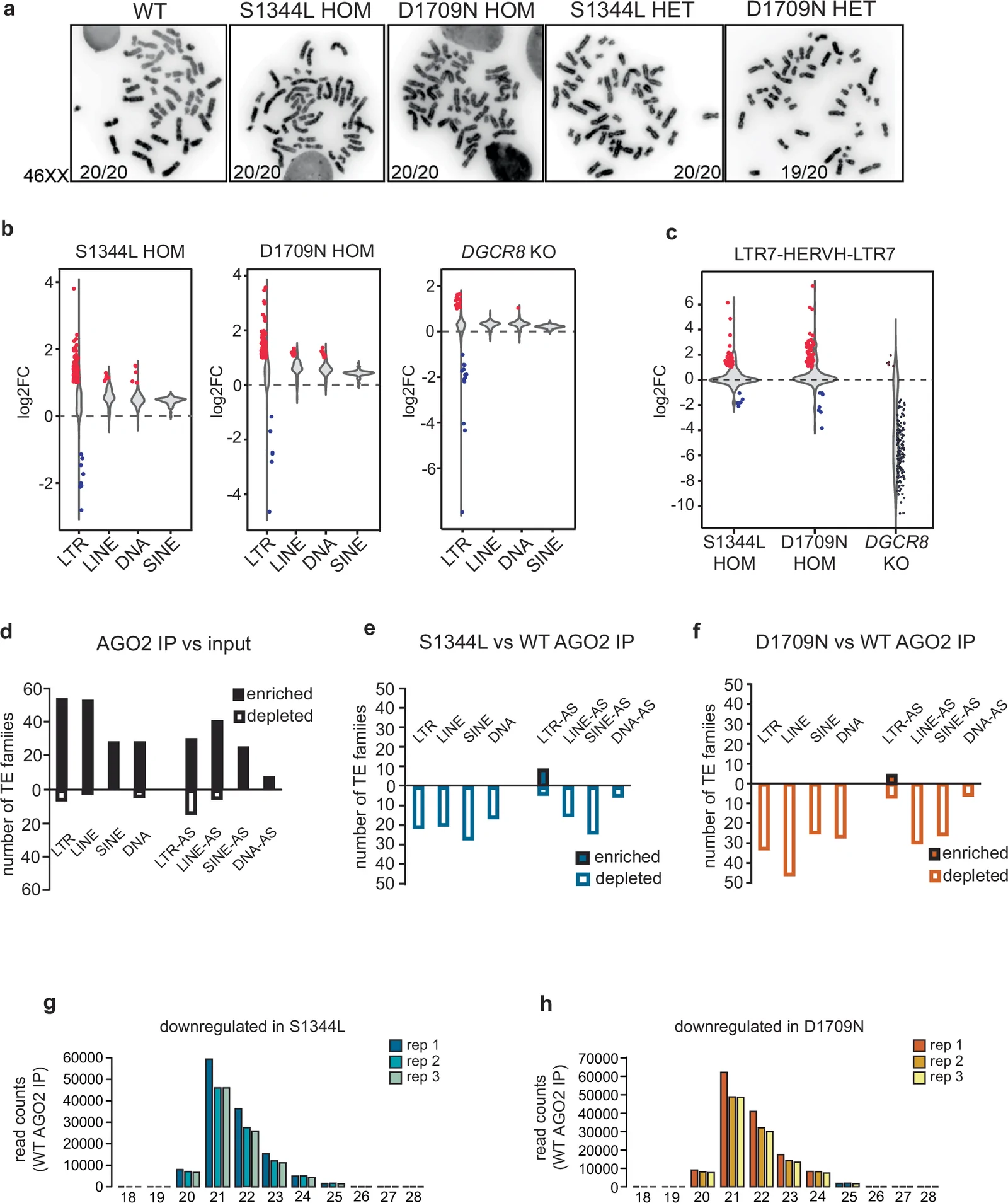
*DICER1* mutations and genome integrity. **a** Representative images of metaphase spreads (*n* = ~20) from PA-1 (WT) and the four *DICER1* mutant cell lines. Counts represent number of spreads with 46XX chromosomes **b** Violin plots for differential expression analyses of LTR, LINE, SINE retrotransposons and DNA transposons, in S1344L HOM (left), D1709N HOM (middle) and DGCR8 KO (right) PA-1 cells versus WT. Significantly upregulated families (red), and downregulated (blue) are shown as individual dots by two-sided Wald test, *p*-adj<0.05. **c** Violin plots for differential expression analysis of defragmented LTR7-HERVH-LTR7 elements. Each dot represents a unique locus of significantly upregulated (red) and downregulated (blue) loci, by two-sided Wald test, *p*-adj < 0.05. **d** Number of TE families that are differentially enriched in the AGO2 immunoprecipitation (IP) compared to the small RNA input of WT cells, classified into LTR, LINE, DNA and SINE. Upregulated (enriched) families are represented as positive counts, downregulated or depleted as negative counts. Reads mapping to all copies of each TE family were analysed separately by sense and antisense (AS) strands (**e**, **f**) Number of differentially enriched TE families in the S1344L AGO2 IPs vs WT cells (**e**) and D1709N AGO2 IP vs WT (**f**) (**g**, **h**) Length distribution of all small RNA reads from WT AGO2 IP that map to TE families that are downregulated in S1344L (**g**) and D1709N (**h**), shown as depleted in (**e**, **f**). Reads from the three replicates (rep1, 2 and 3) of WT-AGO2 IP are shown separately. *X*-axis denotes length of the small RNA read in nucleotides (nt). *Y*-axis shows reads counts.

In addition to miRNA production, mammalian DICER has been reported to be involved in the maintenance of genome stability as well as chromosome segregation during cell division^5,6,9–11^. Most of these studies have been performed in cells where DICER protein is absent, but the specific role of the endonucleolytic activity on these functions has not yet been explored. To study genome stability, we performed metaphase spreads of all the *DICER1*-mutated PA-1 cell lines. PA-1 cells are a female diploid human embryonic teratocarcinoma cell line (46, XX), and all mutants displayed the same chromosome count, indicating that *DICER1* mutations are not associated with increased genome instability or improper chromosome segregation (Fig. 5A).

Mammalian DICER has also been proposed to be involved in the generation of endo-siRNAs or small RNAs with silencing properties against repetitive sequences^12^. To test if this function could be relevant in DRTP, we first assessed transposable element (TE) expression in *DICER1* mutant cells compared to WT using the total RNA high-throughput sequencing datasets. For this purpose, we used an in-house annotation of TEs, in which fragmented elements were merged into full-length copies (see “Methods”). These analyses revealed that both S1344L and D1709N mutations resulted in a general derepression of all types of TEs, i.e., both retrotransposons (including LTR, LINEs and SINEs) and DNA transposons (Fig. 5b). LTRs, including endogenous retroviruses, such as the ERV1 subfamilies, displayed the most dramatic changes (Supplementary Fig. 6A, B). To determine whether this was an indirect effect driven by alterations in miRNA levels, we measured TE expression in *DGCR8* knockout (KO) PA-1 cells, as these lack most canonical miRNAs^32^. This comparison revealed that DGCR8 loss did not result in such a profound general upregulation of TEs as in the DICER mutant cells (Fig. 5b and Supplementary Fig. 6C). More detailed analysis revealed that for some TE types, DGCR8 and DICER mutant cells resulted in opposite behaviours. For instance, the human endogenous retrovirus H (HERVH) was specifically upregulated in DICER mutant cells but not *DGCR8*KO, suggesting a miRNA-independent regulation for this TE (Fig. 5c and Supplementary Fig. 6C). Although TEs are generally silenced in somatic adult cells, HERVH is typically expressed in human early development, and it can be subdivided into several classes depending on the LTR7 sequence^33^. Intriguingly, the most upregulated subfamily in DICER mutant cells were the LTR7up1/2 (Supplementary Fig. 6D), which are highly expressed in pluripotent cells and required to maintain pluripotency^34,35^.

We next explored the mechanisms by which DICER could suppress TE expression. Our results suggested that this regulation was miRNA-independent, although we cannot totally rule out this possibility as DICER mutant cells also display miRNA expression changes that may influence TE expression. Alternatively, TE dysregulation could be explained by the miRNA-independent roles of DICER, including the generation of small RNAs or siRNAs against repeats, thus silencing their expression. To test this possibility, we first assessed if AGO2 was associating with small RNAs derived from TEs. For this, we compared small RNAs mapping to repeats in AGO2 IP versus the input (small RNA dataset) (Supplementary Fig. 6E). We confirmed that small RNAs mapping to both sense and antisense strands of all types of transposons were detected, including LTR, LINE, SINE and DNA (Fig. 5D). TE-derived small RNAs had an average length of 22 nt, in the small RNA-seq datasets that was further enriched after AGO2 immunoprecipitation (Supplementary Fig. 6E). Next, we compared if the association of these small RNAs was altered in DICER mutant cells by comparing the AGO2 IP datasets from WT vs S1344L and D1709N cells. This analysis revealed a reduction in loading of both sense and antisense small RNAs mapping against all types of TEs in both DICER mutant cell lines (Fig. 5e, f). Similar results were obtained with small RNA and AGO2 iCLIP datasets derived from homozygous S1344L hESCs^36^ (Supplementary Fig. 7). Similar to miRNAs, the D1709N mutant seemed to have a more pronounced effect on the loss of TE-derived small RNAs than S1344L, especially for LTRs and LINEs (Fig. 5e, f). For instance, 23 families of LTRs displayed a significant loss of AGO-2-associated small RNAs in S1344L mutant, versus 38 families in D1709N. For LINEs, 33 families displayed significant AGO2-associated small RNA loss in S1344L versus 74 families in D1709N (Fig. 5e, f). The average size of the differentially expressed TE-derived small RNAs in both S1344L and D1709N was 21–23 nt, which is in consistent with production by DICER processing (Fig. 5g, h).

All these together suggest that mutations of the endonucleolytic function of DICER alter loading in AGO2 of sense and antisense small RNAs that are complementary to transcripts derived from TEs, potentially driving their post-transcriptional gene silencing.

### TE and 3p-miRNA upregulation in *DICER1*-mutated tumours

**Fig. 6:**
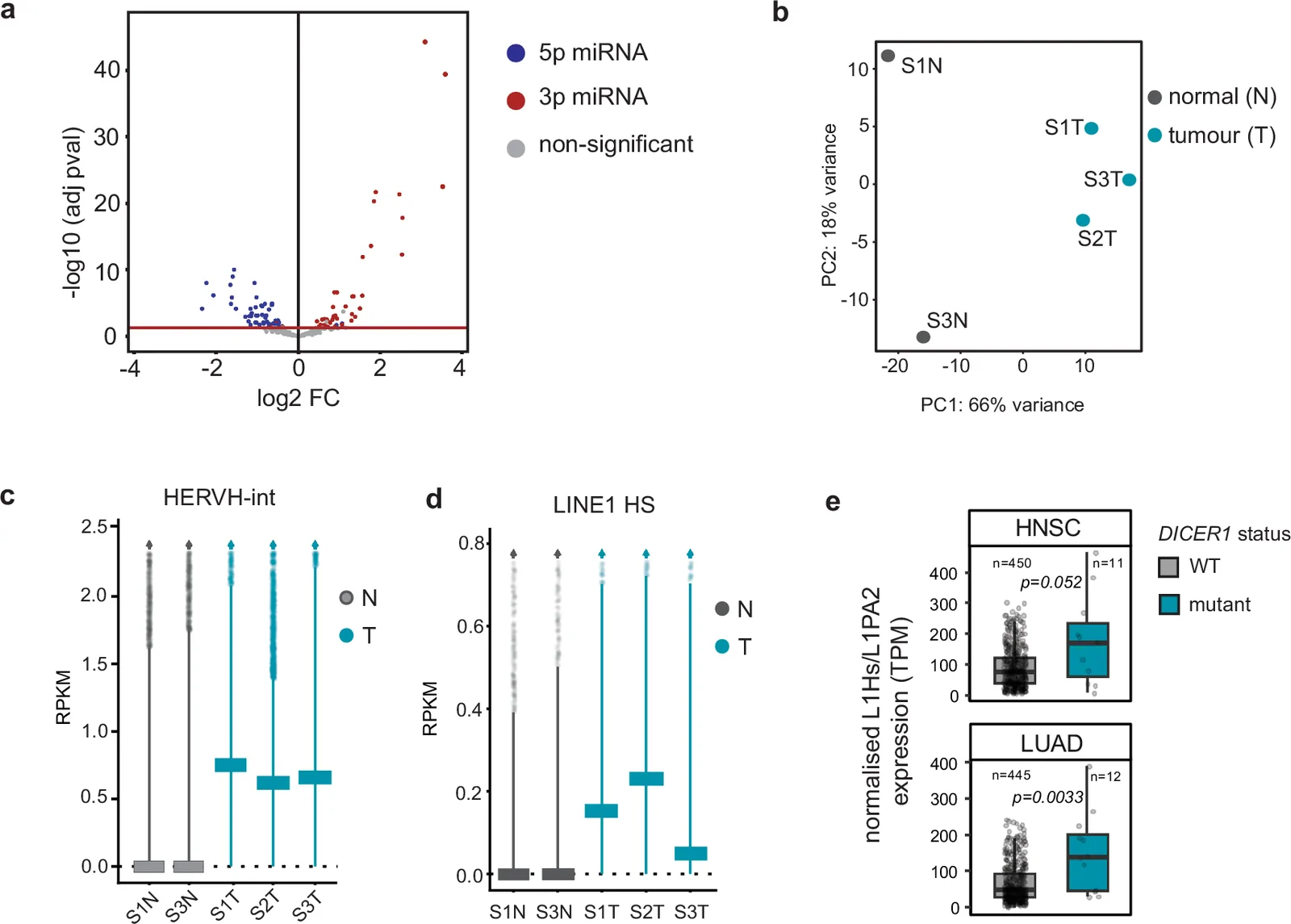
Molecular confirmation in *DICER1*-mutated tumours. **a** Volcano plot showing the log2 fold change of significantly dysregulated 5p- and 3p-miRNAs (two-sided Wald test *p*-adj < 0.05) in *DICER1* tumour (UCEC) samples (*n* = 14), compared to non-*DICER1* mutated (UCEC) samples (*n* = 367). **b** PCA plot for high-throughput RNA-sequencing data of three *DICER1*-mutated tumour samples (S1T, S2T and S3T) and 2 healthy tissues (S1N and S3N) each represented by a single biological sample. **c**, **d** Expression (RPKM) for HERVH-int and LINE 1 HS loci across tumours and healthy samples as designated in (**b**). Median expression is represented by a rectangle and vertical line extends to the 25th and 75th percentiles. Arrowheads indicate points beyond the plotting range. **e** Normalised expression in transcripts per million (TPMs) for active LINE-1 copies (L1Hs and L1PA2) in HNSC (head and neck squamous cell carcinoma) and LUAD (lung adenocarcinoma) with or without *DICER1* mutations (WT and mutant). Number of tumours (*n*) is included at the top of each category. Centre line in box plot indicates median, lower and upper box bounds indicate the 25th and 75th percentile, whiskers extend to the most extreme data points within 1.5 × IQR (interquartile range) of box hinges. Outliers (values below Q1 − 1.5 × IQR or above Q3 + 1.5 × IQR within each group) were excluded from both plots and statistical analysis. Significance was calculated using Wilcoxon rank-sum test.

The results derived from our studies in cell lines suggest that DRTP is not only characterised by a loss in miRNAs, but also a gain of specific 3p-miRNAs and increased expression of TEs. We next investigated if these molecular phenotypes were also observed in *DICER1*-mutated tumours. To this end, we first compared 5p and 3p miRNA expression in TCGA UCEC (uterine corpus endometrial carcinoma) tumour datasets containing *DICER1* biallelic mutations, as defined by ref. ^24^. This confirmed our previous findings in cell lines and showed that *DICER1*-mutated tumours also displayed a general decrease of 5p-miRNA levels and increased 3p-miRNA abundance compared to non-mutated tumours (Fig. 6a).

We next investigated if the increased TE expression phenotype was also observed in DRTP tumours. To this end, we performed total RNA high-throughput sequencing from three different tumours containing *DICER1* mutations, two with mutations typically found in DRTP (E1705K), and one with a frameshift mutation resulting in a truncated protein lacking both RNAse III motifs (Fig. 6b and Supplementary Fig. 8). We observed that, independently of the type of mutation, inactivating DICER resulted in increased average expression of both HERVH INT (the internal region between the LTR7 sites) and the most active LINE-1 element, LINE-1 Hs (Fig. 6c, d). In agreement with these results, the expression of LINE-1 Hs and LINE PA-2, the two active LINE-1 elements in humans, was increased in *DICER1-*mutated HNSC (head and neck squamous cell carcinoma) and LUAD (lung adenocarcinoma) samples from the TCGA repository (Fig. 6e), suggesting that independently of the type of *DICER1* mutation, inactivation of DICER results in increased TE expression.

Altogether, we propose that dual functions of DICER in miRNA biogenesis and TE expression are critical to understanding the molecular mechanisms by which *DICER1* mutations can cause DRTP.

## Discussion

Mutations in the *DICER1* gene are associated with DRTP. However, DICER is not the only small RNA biogenesis factor that has been found to be mutated in cancer. DICER, along with a number of key components of the small RNA biogenesis pathways, such as DROSHA and DGCR8, are found to be mutated in Wilms tumours, the most prevalent paediatric kidney malignancy^37–40^. Similar to our *DICER1*-mutated cell models, *DROSHA* mutations are also associated with increased mesenchymal phenotypes^41^. However, in tumours, *DROSHA* is typically only mutated in a single allele that behaves as a dominant negative. Often, these hotspot mutations affect the RNase IIIb domain, disrupting the 5′ end cleavage of the pri-miRNA^38,40^. In contrast, mutations in *DGCR8* usually affect both alleles and interfere with its dsRNA-binding activity, leading to impaired pri-miRNA processing^37,39,41^. Given that DGCR8 and DROSHA function upstream of DICER, mutations in these factors result in unproductive processing, leading to depletion of both 5p and 3p miRNAs. This phenomenon has been verified in cell models and tumours carrying *DROSHA* mutations^38,39,42^.

The specific loss of 5p miRNAs upon *DICER1* mutations had been reported in earlier studies^42,43^. While initial research suggested that 3p miRNAs were not affected, more recent studies observed an increase in 3p miRNA abundance in tumours^24,44,45^. Only recently has this increase in 3p miRNA been confirmed in cell models recapitulating DRTP mutations, in agreement with our findings. Jee et al. and Malagobadan et al. confirmed the increased abundance of 3p passenger miRNA loading into AGO2 using a mutant of the RNase IIIa in human stem cells and a mutant of the RNase IIIb in mouse cells, respectively^36,46^. However, a direct comparison between the consequences of human RNase IIIa and IIIb domain hotspot mutations within the same genetic background was not performed in these studies. Our results indicate that whilst both mutations result in a similar increase of 3p miRNAs, the D1709N mutation causes a more pronounced loss of 5p miRNAs compared to S1344L. This is consistent with the structure of human DICER, where both residues contribute to placing the dsRNA substrate in the correct position in the active site of the enzyme^30^. While S1344 is structurally part of the active site for 5p miRNA, unlike D1709, it does not participate in metal-ion binding required for catalysis, suggesting that some (low) level of 5p processing might still be possible in these mutants.

Supporting the relevance of 3p miRNA dysregulation in *DICER1*-mutated tumours, our findings demonstrate that the expression of predicted targets for these miRNAs is significantly altered. We have also confirmed these findings using 3p miRNA luciferase-based reporters. This suggests that, unlike mutations in DROSHA and DGCR8, the molecular phenotypes associated with DRTP mutations originate from both the loss and gain of specific miRNAs. It will be interesting to explore whether tissues commonly affected by *DICER1* mutations, such as the pleura in the lungs, ovaries, kidneys or thyroid, exhibit similar biases towards 5p or 3p guides^47^. For instance, are tissues dominated by 5p-guides more susceptible to *DICER1* mutations? The cells used in this study are 3p-dominated; ~40% of the total miRNA content of PA-1 cells derived from the miR-302a-3p and miR-302b-3p miRNAs. Given that 3p guides are not affected, cells with 3p-dominant profile could experience less severe impacts of *DICER1* inactivation. Conversely, cell types and tissues that are 5p-dominated will likely experience a more dramatic change in their miRNA repertoire upon *DICER1* inactivation.

In addition to miRNAs, we have explored the relevance of less well-characterised functions of DICER in the context of DRTP mutations. DICER is known to play roles in maintaining genome stability and ensuring proper chromosome segregation^5,6,9–11^, and malfunction in both can lead to aneuploidy. Our results showed, however, that endonucleolytic inactivation of DICER did not result in the types of chromosomal abnormalities reported in cells lacking *DICER1*. Our results are in agreement with previous reports that show that genome instability is not a typical feature of human *DICER1*-related tumours^47,48^. Unfortunately, we were unable to obtain full knockouts of *DICER1* in PA-1 cells to confirm if aneuploidy is only observed upon full deficiency. It is possible that the complete inactivation of *DICER1* is lethal in PA-1 cells, as previously observed in human embryonic stem cells^26^. Nevertheless, we did obtain *DICER1*^*+/−*^ cells, and these cells did not show altered miRNA levels, suggesting that *DICER1* is not haploinsufficient in human cells, as previously demonstrated for mouse *Dicer*^8^. The observation that human *DICER1* is not haploinsufficient may explain why germline carriers, where only one allele is inactivated, are viable, and they do not develop the disease until the second mutation occurs. More recently, *Dicer1*^*+/−*^ tumour mouse models have shown that heterozygous loss of *Dicer1* can affect the onset and penetrance of tumour development^49^.

Previously, mouse *Dicer1* had been implicated in the repression of transposable elements (TEs)^11,16^. We confirmed these findings in the context of human DICER endonucleolytic inactivation, showing that DICER represses both retrotransposons and DNA transposons. We hypothesise that some of this repression may be driven, in part, by the production of small RNAs or endo-siRNAs. SiRNAs can be generated by DICER cleavage of endogenous RNA substrates, including double-stranded RNA (dsRNA). This activity is typically observed only in pluripotent embryonic stem cells and oocytes^19,20,50^. Interestingly, these two cell types, as well as embryonic teratocarcinoma cells, are devoid of the type I interferon (IFN) response, meaning they cannot produce IFN in response to cytoplasmic dsRNA accumulation, in contrast to somatic cell types^4,51,52^. Notably, PA-1 are derived from an ovarian embryonic teratocarcinoma, suggesting their IFN response is attenuated and that they can tolerate a certain level of endogenous dsRNA. In line with a potential role for endo-siRNAs in the control of TEs, we observed that *DICER1* mutations affect the association of antisense small RNAs against various TE, including ERVs. The HERVH upregulation seems to be miRNA-independent, since cells lacking all canonical miRNAs do not display upregulation of these elements, confirming previous findings for DGCR8-deficient human embryonic stem cells^53^. Importantly, the increased LTR7-HERVH and LINE-1 expression was also confirmed in *DICER1*-mutated tumours. Intriguingly, LINE-1 overexpression is a hallmark of cancer, and HERVH reactivation has also been observed in both liver metastases and primary colorectal cancers^54–56^. Although the role that HERVH plays in cancer development, the immune status, and its value as a predictive prognostic marker is still to be investigated.

We hypothesise that the upregulation of LTR7 in *DICER1* mutant cells has two potentially important consequences for our understanding of DRTP. First, LTR7-HERVH, particularly the LTR7up1/up2 subfamilies, are the most highly expressed copies in human embryonic stem cells and are essential for the maintenance of pluripotency^35,57–59^. The parallels between pluripotent embryonic cells and cancer cells are striking: both undergo EMT transitions and require high self-renewal capacity^60^. Furthermore, the reactivation of LTR7 elements in cancer cells can lead to the generation of novel transcripts. For example, the activity of the LTR7 promoter in human embryonic stem cells results in the production of chimeric transcripts^59,61^. In the context of cancer, this property could be exploited to develop T-cell therapies against neo-antigens. For instance, *env* HERVH-encoded proteins have been used to train cytotoxic T cells to target colorectal carcinoma cells^62^. Additionally, the reactivation of LTRs can also result in the expression of oncogenes, in a process called onco-exaptation^63^. Exploring these dynamics in the context of *DICER1* mutations could revolutionise our understanding of these tumours and their potential treatments.

## Methods

### Ethics

Compliance with all relevant regulations regarding human participants was maintained throughout this study, and the research was conducted in accordance with the principles of the Declaration of Helsinki. Ethical approval for accessing archival donor tissue was issued by the Newcastle and North Tyneside 1 Research Ethics Committee via the NovoPath Biobank (Newcastle Upon Tyne Hospitals NHS Foundation Trust). Project references CEPA182 and CEPA223 (“Understanding the molecular consequences of Dicer mutations in *DICER1*”) were approved by the Hospital Biobank Access Committee. Living donors provided informed consent, and patient confidentiality was protected via sample de-identification or pseudonymisation.

### Cell lines

Human PA-1 cells (ATCC CRL-1592) were cultured in Minimum Essential Medium (MEM) with GlutaMAX (Gibco) supplemented with 20% heat-inactivated fetal bovine serum (FBS, Gibco), 50 µg/ml Gentamicin (Gibco) and 0.1 mM MEM Non-Essential Amino Acids (Gibco) at 37 °C with 5% CO_2_. Routine Mycoplasma testing was performed.

### Generation of CRISPR/Cas9 targeted clones

CRISPR RNA guides (crRNAs) were designed to target exon 21 or exon 24 close to the respective amino acid residues S1344 or D1709 in the RNase III a or b domains of DICER using the Cas9 design option from CRISPR Design Tool (https://chopchop.cbu.uib.no/) along with repair templates with the respective point mutations and silent mutations to remove PAM sites and introduction of unique restriction sites to aid screening to identify targeted clones.

crRNA-S1344 (5′-CACTTACCCTGATGCGCATG-3′),

crRNA-D1709 (5′ AGTCCAAAATCGCATCTCCC-3′)

Repair template-S1344L

(5′CTGCAAACCACTTTCAGGCACACTGAATAATTAACTGCTCAAAATAAAAAAATCATCTCTTACCTTTTTGCTTCTCATATATAAAAGGCGCCCTTCATGCGCATCAGGGTAAGTGCAAAATAGATATGTGG-3′).

Repair template -D1709N

(5′CTTCGGATCCCCTCAGATTGTTACCAGCGCTTAGAATTTCTGGGAAATGCAATATTGGACTACCTCATAACCAAGCACCTTTATGAAGACCCGCGGCAGCACTCCCCGGGGGTCCTGACAGACCTGCGGTCTGCCCTGG-3′)

Equimolar amount of either crRNA-S1344 or crRNA-D1709 (245pmol, IDT) where incubated with tracrRNA (245 pmol, IDT) in 25 µl nuclease-free buffer at 95 °C for 5 min and annealed by allowing to cool to room temperature (RT). These guides were then incubated with 25 µg Alt-R® S.p. Cas9 Nuclease V3 (IDT) in 22.5 µl buffer T (Neon Transfection System, ThermoFisher Scientific) for 20 minutes at 37 °C prior to electroporation. PA-1 cells (1.2 × 10^6^ per transfection) were resuspended on 80 µl of Buffer T (Neon Transfection Systems), the Cas9*/*gRNA mixture and the corresponding repair template (300 pmol, IDT) were added to each tube. Electroporation was performed with three pulses of 1600 V and 10 ms. Cells were transferred to T25 cm flask overnight before isolating single-cell clones by limiting dilution in 96-well plates. The Platinum Direct PCR Universal Mastermix was used to screen for edited clones as per the manufacturer’s instructions using the following PCR primer pairs.

S1344L-F1 5′GAAATACCCGTGCAACCAA 3′

S1344L-R1 5′ TGTAAGCCAAGACGTACCCTC 3′

D1709N-F1 5′ CGGCCCTGGTTTCACTTAA 3′

D1709N-R1 5′ GCAGACAAGTTCAAGAGGCC 3′

The resulting PCR product (uncut) or diagnostic restriction digest was performed prior to running on 1.5% agarose gel and visualisation to identify targeted clones. The PCR product from targeted clones where either directly sequenced using Nanopore sequencing (Plasmidsaurus) or cloned into pGEM-T Easy vector (Promega) and confirmed by Sanger sequencing. The homozygous point mutant *DICER1*^S1344L/S1344L^, *DICER1*^D1709N/D1709N^ and the compound heterozygous mutant *DICER1*^D1709N/FS^ were generated with a single round of targeting. To generate the *DICER1*^*S1344L/FS*^ compound heterozygous mutant a second round of targeted was carried out using a heterozygous cell line *DICER1*^*S1344L/+*^ which harboured the S1344L point mutation on one allele and PAM site silent mutation and the other allele was wild type.

### Metaphase spreads

For karyotype analysis, each cell line was grown to approximately 80% confluency in a T75cm flask and treated for 30 min at 37 °C with colcemid (KaryoMAX Colcemid, Gibco), trypsinised and cells ruptured using a hypotonic solution (0.075 M KCl). The cell nuclei were then fixed using a 3:1 methanol/acetic acid solution and dropped on microscope slides before counting metaphase chromosomes (20 metaphase spreads per cell line were counted).

### Western blotting

Total cell lysates were prepared by lysing cell pellets in RIPA buffer supplemented with cOmplete EDTA-free protease inhibitor cocktail tablets (Roche), as per manufacturer’s instructions and centrifugation (13,000 × *g* for 20 min at 4 °C) to clarify the extracts. The BCA assay (Pierce) was used to determine protein concentration and samples stored at −80 °C until required. Proteins were separated on NuPage Novex Bis-Tris 4–20% Tris-Glycine protein gels (Invitrogen) and transferred to Immobilon-FL PVDF membrane (Sigma) by wet transfer. Membranes were blocked for 1 h at RT with 5% milk powder in TBS-T (0.1% Tween-20, Sigma) and incubated with primary antibodies overnight at 4 °C. Primary antibodies used were mouse anti-DICER (1:1000 dilution, Clone 13D6, Cat # AB13D6, Abcam), rabbit anti-AGO2 (1:1000 dilution, Clone C34C6, Cat # 2897S, Cell Signalling) and mouse anti-Tubulin (1:4000 dilution CP06, Millipore). Membranes were washed in TBS-T and incubated with secondary antibodies (Goat anti-rabbit IgG [H + L] or Goat anti-mouse IgG [H + L]) for 1 h at RT and washed again before visualisation of protein bands using Odyssey CLx Scanner (LiCor). Protein bands were quantified using ImageJ (v1.53q) software and expression levels calculated normalized to tubulin.

### Growth rate/Proliferation assays

Cell growth rates were measured by seeding 1 × 10^5^ cells in triplicate in 6-well dish (day 0) and cell counts taken on the indicated days (3, 6, 9, 12 days) using a haemocytometer and cell counter before re-seeding.

### Metabolic activity assay

Metabolic activity was measured over time in the different cell lines by seeding cells in a 96-well plate (2 × 10^3^ cells per well) and measuring ATP levels using the CellTiter-Glo assay (Promega) at 7 time points (0, 6, 24, 30, 48, 54, 72 h). Luminescence was recorded using Varioskan Flash (ThermoFisher Scientific) with an integration time of 1000 ms per well.

### Colony formation assay

Triplicate 6-well dishes were seeded (5 × 10^2^ cells/well) and grown for 7 days, then washed with PBS, fixed with 70% ethanol and stained with 0.1% Crystal Violet (Sigma) before washing in water to remove background staining. Celigo image cytometer (Nexcelom Bioscience) was used to scan crystal violet-stained colonies in the brightfield channel. Software version 5.6.0.0 was used to calculate the number and size of colonies using the “Colony” application.

### Wound healing assay

Cells were seeded in triplicate in 6-well dishes to give a confluent monolayer the following day. A scratch was made in the centre of each well using a 20 μl plastic tip, the wound was gently washed with PBS to remove residual detached cells and fresh growth media added. Images were taken using EVOS M7000 (×2 Objective AMEP431, transwell) at 0, 12, 24 and 36 h post scratch. Quantification of the wound closure was measured using Image J software.

### Immunoprecipitation (IP)

Protein G beads (Invitrogen) were prepared by conjugating 50 μl beads to 5 μg of anti-rat AGO2 antibody (Clone 11A9, Cat # MABE 253, Millipore) or rat IgG control per sample. The beads were equilibrated in lysis buffer (50 mM Tris-HCl pH 7.8, 300 mM NaCl, 1% Triton ×-100, 5 mM EDTA, 10% Glycerol) prior to the addition of total cell lysates and incubated at 4 °C overnight with rotation. The beads were washed as follows: Low Salt (LS)-wash (50 mM Tris-HCl pH 7.8, 300 mM NaCl, 5 mM MgCl_2_, 0.5% Triton ×-100, 2.5% glycerol), twice and High Salt (HS)-wash (50 mM Tris-HCl pH 7.8, 800 mM NaCl, 5 mM MgCl_2_, 0.5% Triton ×-100, 2.5% glycerol). For downstream protein analysis, the IP-protein fraction was eluted in LDS sample buffer (Life Technologies) containing 100 mM DTT (Sigma) at 70 °C for 10 min with shaking. For downstream RNA analysis, the IP-RNA fraction was resuspended in QIazol (700 µl) and RNA extracted using the miRNeasy mini kit (QIAGEN) following manufacturer’s instructions.

### miRNA quantification by RT-qPCR

Extracted RNA from each cell line was reverse transcribed using miRCURY LNA miRNA PCR starter kit (Qiagen, Cat.No.339320) as per manufacturer’s instructions. The synthesised cDNAs were diluted (1:60) as recommended and qPCR carried out using small RNA miRCURY qPCR primer assays (Qiagen). Both 5p and 3p miRNAs were quantified for hsa-miR-17,18a, 93, 7 and SNORD 44 was used as a normaliser.

### 5p- and 3p-miRNA luciferase-based sensors

Three copies of complementary 5p- or 3p-miRNA sequences, separated by a 4 base pair linker, were designed and synthesised by IDT for human miR-17 and miR-7 with Xho1 and Not1 sites.

miR-17-5p Fwd

(5′TCGAGCTACCTGCACTGTAAGCACTTTGTTATCTACCTGCACTGTAAGCACTTTGTTATCTACCTGCACTGTAAGCACTTTGGC 3′)

miR-17- 5p Rev

(5′GGCCGCCAAAGTGCTTACAGTGCAGGTAGAATACAAAGTGCTTACAGTGCAGGTAGAATACAAAGTGCTTACAGTGCAGGTAGC 3′)

miR-17-3p Fwd

(5′TCGAGCTACAAGTGCCTTCACTGCAGTTTATCTACAAGTGCCTTCACTGCAGTTTATCTACAAGTGCCTTCACTGCAGTGC 3′)

miR-17-3p Rev

(5′GGCCGCACTGCAGTGAAGGCACTTGTAGAATAACTGCAGTGAAGGCACTTGTAGAATAACTGCAGTGAAGGCACTTGTAGC 3′)

miR-7-5p Fwd

(5′TCGAGAACAACAAAATCACTAGTCTTCCATTATAACAACAAAATCACTAGTCTTCCATTATAACAACAAAATCACTAGTCTTCCAGC 3′)

miR -7-5p Rev

(5′GGCCGCTGGAAGACTAGTGATTTTGTTGTTTAATAGGAAGACTAGTGATTTTGTTGTTTAATAGGAAGACTAGTGATTTTGTTGTTC 3′)

miR-7-1-3p Fwd

(5′TCGAGTATGGCAGACTGTGATTTGTTGTTATTATGGCAGACTGTGATTTGTTGTTATTATGGCAGACTGTGATTTGTTGGC 3′)

miR-7-1-3p Rev

(5′GGCCGCCAACAAATCACAGTCTGCCATAAATACAACAAATCACAGTCTGCCATAAATACAACAAATCACAGTCTGCCATAC 3′)

DNA duplexes were directionally cloned into pre-digested and purified psiCHECK2-Xho1/Not1 and sequence verified (Plasmidsaurus).

For luciferase assays WT and *DICER1* mutant cells (1.5 × 10^5^) were seeded in duplicate into 24-well plate and transfected the following day with either psiCHECK2-miR-17-5p, psiCHECK2-miR-17-3p, psiCHECK2-miR-7-5p or psiCHECK2-miR-7-3p using Lipofectamine 2000 (Invitrogen) using manufacturer’s recommendations. Firefly and *Renilla* luciferase quantification were performed 48 h post-transfection using the Dual-Luciferase Reporter Assay System (Promega) and Varioskan Flash (Thermo Scientific) following manufacturer’s recommendations.

### Small RNA high-throughput sequencing and analysis

RNA was extracted from WT and *DICER1*-mutant cell lines in triplicate using the miRNeasy mini kit for both input (total cell extracts) or AGO2-IPs. RNA recovered was quantified using the Qubit 2.0 Fluorometer (ThermoFisher Scientific, Q32866) and the quality assessed on the Agilent 2100 Electrophoresis Bioanalyser Instrument (Agilent, G2939AA). Library preparation and sequencing was carried out by the Clinical Research Facility, Edinburgh. For small RNA sequencing libraries, 500 ng of total RNA was used from each of the samples and libraries prepared using the QIAseq miRNA library kit 96 (QIAGEN, Cat #331505) and the QIAseq miRNA 96 Index Kit IL UDI-E (Cat #331655) according to the manufacturer’s protocols. Purified libraries were then amplified for 13 cycles of PCR with unique 10 bp dual index (UDI) sequences to allow multiplexing on a single sequencing run. Amplified, indexed libraries were purified and size-selected with QMN beads to enrich for library fragments containing miRNAs. Sequencing was performed on the NextSeq 2000 platform (Illumina Inc, #20038897) using NextSeq 1000/2000 P1 Reagents (100 Cycles) (#20074933). Sequencing was single-end 1 × 75bp to allow coverage of the UMI sequence.

Small RNA-Seq data were processed as follows. FastQC and MultiQC were used to evaluate the quality of the raw reads (https://www.bioinformatics.babraham.ac.uk/projects/fastqc/)^64^. Reaper was used to remove the 3՛ adapter sequences, and distinct sequences were collapsed using tally, selecting those between 20 and 25 nt for differential expression analysis, or 18–40 nt to explore read length distribution, and keeping track of the counts of each read^65^. Unique reads were mapped against the human genome (GRCh38), using bowtie1 v1.3.1^66^, allowing 1 mismatch and reporting all possible locations (-v 1 –all –best –strata). A Python script was then used to distribute each read’s counts evenly between all mapping locations. The remaining analyses were conducted within the *R* environment for statistical computing (https://www.r-project.org/). FeatureCounts was used to quantify overlapping reads^67^. Differential expression tests were performed within the edgeR framework, with quasi-dispersion estimates, and using glmTreat to test against a minimum fold-change threshold of 2 and a Benjamini–Hochberg corrected FDR < 0.01^68^. Normalisation factors were calculated manually using tRNAs as the reference annotation. Plotting used a combination of base *R*, ggplot2, ggside and ggrepel (https://ggplot2-book.org/). A custom genome annotation was developed, compiling protein-coding, lncRNA and tRNA annotation from Gencode r49^69^, microRNAs from miRBase r22.1^70^ and MirGeneDB r3.0^71^, mirtrons from mirtronDB v2^72^, and repeats as described under “Transposable elements analysis” below. Care was taken to remove redundancy due to overlaps, giving preference to known microRNA annotation. To explore read length distribution, TE coordinates were extracted from our custom annotation file, and bedtools intersect was used to extract all reads mapping with these coordinates from each BAM file^73^. Read lengths were tabulated and plotted using R.

### Modelling of DICER mutations

Coordinates from human DICER bound to pre-let7 (PDBid 7XW2^30^) were mutated in silico, using the mutation commands in Coot 0.9.6^74^, selecting a common rotomer for each mutant. For D1709N, the rotomer most closely matching the sidechain in the experimental structure was used. Images of the mutant residues superimposed on the native structure were generated using Pymol 3.1.6.1 (https://www.pymol.org.).

### Total RNA high-throughput sequencing and analysis

Libraries for total RNA sequencing were prepared using the NEBNext Ultra 2 Directional RNA library prep kit (Illumina, E7760) and the NEBNext rRNA Depletion kit (Human/Mouse/Rat) (Illumina, E6310) according to the provided protocol. Sequencing was performed on the NextSeq 2000 platform (Illumina, 20038897) using NextSeq 2000 P3 Reagents (200 Cycles, 2 × 100bp) (20040559).

Total RNA-sequencing runs were processed with the following pipeline. Gene based read counts and TPMs were retrieved for each sample after mapping over human genome GRCh38/hg38 using Gencode39 annotation (Ensembl-based) with STAR v2.7.9a^75^ with default parameters. Statistical inference of regulated genes of one comparison of grouped *DICER1* mutants vs WT samples were calculated with the DESeq2 v1.34 ^76^ R library by setting the contrast parameter and weighted equally to the log2 fold changes of the four *DICER1* mutants. Differentially regulated genes were retrieved with an FDR cut-off of 0.05 obtained with the Benjamini & Hochberg correction.

### Total cell proteomics

Total protein cell extracts were prepared by lysing cells in ice-cold RIPA buffer (50 mM Tris/HCl pH 7.4, 150 mM NaCl, 1% Triton-×100, 0.5% Sodium Deoxycholate, 0.1% SDS supplemented with cOmplete protease inhibitor cocktail tablets—Roche). Lysates were incubated on ice for 20 min. Insoluble cell debris was pelleted by centrifuged at 13,000 × *g* for 20 min at 4 °C and the clarified total cell protein supernatant was saved as total cell extracts. Protein concentration was measured using BCA protein assay (ThermoFisher Scientific, 23225) as per manufacturer’s instructions. Four replicates were generated for each of the cell lines (WT, S1344L HOM and D1709N HOM). For each individual replicate, proteins were purified using the SP3 magnetic bead method with a 1:1 mixture of Sera-Mag E3 and E7 beads. Protein samples were reduced with 10 mM dithiothreitol (DTT) for 30 min at 56 °C and alkylated with 50 mM iodoacetamide (IAA) for 30 min in the dark at room temperature, followed by quenching with 10 mM DTT. Beads were added at a ratio of 10 µg beads per 1 µg protein, and protein binding was induced by adding ethanol to a final concentration of 50%. Samples were mixed and incubated briefly for capture, followed by three washes with 80% ethanol and brief air-drying to remove residual solvent. Bound proteins were digested overnight at 37 °C in 50 mM ammonium bicarbonate using trypsin at a 1:50 enzyme-to-protein ratio (w/w). Digestion was stopped by acidification with trifluoroacetic acid (TFA), and peptides were captured on pre-equilibrated C18 StageTips. StageTips were washed to remove contaminants and stored at −20 °C, as described in ref. ^77^. Peptides were eluted in 40 μL of 80% acetonitrile (Fisher Scientific) in 0.1% TFA and concentrated down to 1 μL by vacuum centrifugation (Concentrator 5301, Eppendorf, UK). The peptide sample was then prepared for LC-MS/MS analysis by diluting it to 5 μL by 0.1% TFA.

LC-MS analyses were performed on an Orbitrap Exploris 480™ on Data Independent Acquisition (DIA), coupled on-line, to an Ultimate 3000 HPLC (Dionex, Thermo Fisher Scientific, UK). Peptides were separated on a 50 cm (2 µm particle size) EASY-Spray column (Thermo Scientific, UK), which was assembled on an EASY-Spray source (Thermo Scientific, UK) and operated constantly at 50 °C. Mobile phase A consisted of 0.1% formic acid in LC-MS grade water and mobile phase B consisted of 80% acetonitrile and 0.1% formic acid. Peptides were loaded onto the column at a flow rate of 0.3 μL min^−1^ and eluted at a flow rate of 0.25 μL min^−1^ according to the following gradient: 2–40% mobile phase B in 150 min and then to 95% in 11 min. Mobile phase B was retained at 95% for 5 min and returned back to 2% a minute after until the end of the run (190 min).

MS1 scans were recorded at 120,000 resolution (scan range 350-1650 m/z) with an ion target of 5.0e6, and injection time of 20 ms. MS2 DIA was performed in the orbitrap at 30,000 resolution with a scan range of 350–1200 m/z, maximum injection time of 55 ms and AGC target of 3.0E6 ions. We used HCD fragmentation^78^ with stepped collision energies of 25.5, 27 and 30. We used variable isolation windows throughout the scan range ranging from 10.5 to 50.5 m/z. Shorter isolation windows (10.5–18.5 m/z) were applied from 400 to 800 m/z and then gradually increased to 50.5 m/z until the end of the scan range. The default charge state was set to 3. Data for both survey and MS/MS scans were acquired in profile mode.

The DIA-NN software platform^79^ version 1.9.2. was used to process the DIA raw files and search was conducted against the canonical *Homo sapiens* database (Uniprot, released August 2022). Precursor ion generation was based on the chosen protein database (automatically generated spectral library) with deep-learning based spectra, retention time and IMs prediction. Digestion mode was set to specific with trypsin allowing maximum of two missed cleavages. Carbamidomethylation of cysteine was set as fixed modification. Oxidation of methionine, and acetylation of the N-terminus were set as variable modifications. The parameters for peptide length range, precursor charge range, precursor m/z range and fragment ion m/z range as well as other software parameters, were used with their default values. The precursor FDR was set to 1%.

Differential protein abundance was performed using Perseus (proteinGroups) outputs. Keratins were filtered out as contaminants, proteins missing values (N/A) across all compared samples were also excluded. For relative quantification, protein row intensities were normalised and transformed using the R-package Variance Stabilising Normalisation, VSN (3.74.0)^80^. Missing value Imputation was performed by determining the global minimum value, and imputation was performed only for proteins undetected in all replicates in one experimental condition, while present in the other condition (at least in 2 replicates, ON/OFF). The R package limma (3.62.2)^81^ was used for statistical analysis of the processed protein intensities, applying the empirical Bayesian method moderated *t*-test with *p*-values adjusted for multiple testing using the Benjamini–Hochberg method.

### Transposable elements analysis

Transposable element annotation for hg38 was obtained from the UCSC Genome Browser. A defragmented annotation was obtained by merging individual TE fragments into whole copies using Onecodetofindthemall.pl^82^, and defragmented copies were renamed according to their position and structure. This defragmented annotation was used to perform locus-specific quantification. To specifically analyse the expression of LTR7 subfamilies, we used the recent annotation by ref. ^33^.

TE expression both at family and individual locus level was quantified using TEcount and TElocal tools from TEtranscript toolkit, respectively^83^. First, defragmented TE annotation was indexed for TElocal using script TElocal_indexer provided by TEtranscript developers, and sequencing reads were aligned with STAR v2.7.11b^75^ using parameters recommended by authors in ref. ^83^. Both TEcount and TElocal distributed multimapped reads among all alignments.

Differential expression analysis was carried out using DESeq2^76^, estimating size factors considering only genes and applying lfcShrink function to shrink log2 fold changes. Volcano and violin plots were generated using in-house *R* scripts with ggplot2.

### TCGA analysis

Quantification for isoform microRNA expression was retrieved for TCGA-UCEC tumour samples using TCGAbiolinks package^84^ and downloaded with the GDC Data Transfer Tool. microRNA samples were classified as *DICER1* mutated hotspots (as described in ref. ^24^) and non-*DICER1* mutated samples. Non-*DICER1* mutated samples were further filtered to remove samples with mutations in genes involved in microRNA biogenesis (*DROSHA*, *DGCR8* and *AGO* family genes).

MicroRNAs were assigned to 5p or 3p group according to their mirBase v21 name prefix. Fragments annotated as precursor, stemloop, or unannotated were excluded. Differential expression analysis of *DICER1* mutated tumour samples (*n* = 14) versus non-*DICER1* mutated samples (*n* = 367) was carried out with DESeq2. microRNAs were considered dysregulated if *p*-adj < 0.05.

LINE-1 expression data were obtained from the TCGA pan-cancer analysis by ref. ^85^. Specifically, normalised expression levels (TPM) for LINE1-Hs and L1PA2 elements quantified using L1EM was used directly. Samples were grouped by *DICER1* mutational status using annotations from ref. ^24^.

### miRNA predicted targets and comparison to transcriptomic/proteomic data

Predictions for all human miRNA targets were obtained from Targetscan r8.0^86^, selecting the cumulative weighted context++ scores (CWCS) from the “Summary Counts for all predictions” file. Target genes were mapped to the transcriptomic and proteomic experiments using the official Gene Symbol. To understand if predicted targets were changing as expected, we focused on the 10 most up/downregulated miRNAs from the D1709N or S1344L mutants vs WT sRNA-Seq data (fold-change > 10, FDR < 0.05 and sorted by average CPM). For each of these groups of miRNAs we chose the top 500 targets (according to CWCS) that were also measured in the transcriptomic or proteomic experiment. Assuming that the effect of a gene targeted by upregulated as well as downregulated miRNAs would be difficult to predict, we excluded genes that were also within the top 1000 predicted targets of miRNAs changing in the opposite direction. The remaining genes (<500) were analysed by plotting their logFC cumulative distribution and by performing a one-tailed Wilcoxon Rank Sum test against the genes that were not in any of the predicted target groups. The alternative hypothesis for up(down)-regulated miRNAs was that targets will be down(up)-regulated.

### Human FFPE tissue sequencing

FFPE (formalin-fixed paraffin-embedded) tumour and normal tissue were obtained from NovoPath, Newcastle Upon Tyne hospitals NHS foundation under the relevant ethical approval (see Ethics). RNA extraction, QC, library preparation and RNA-sequencing of FFPE curls (10 × 10 μm) each tumour and normal tissue was performed by Genewiz, Azenta, using their standard RNA-seq paired end (2 × 150 bp) sequencing service NovaSeq XPlus. To quantify TE expression, the same pipeline as for RNA-seq from PA-1 cells was used. TE expression was quantified using TEcount and TElocal tools from TEtranscript toolkit^83^. Standard fragmented TE annotation provided by TElocal was used. All plots were generated using in-house R scripts with ggplot2.

### Reporting summary

Further information on research design is available in the Nature Portfolio Reporting Summary linked to this article.

## Supplementary information

Supplementary Information

Description of Additional Supplementary Files

Supplementary Data 1

Supplementary Data 2

Supplementary Data 3

Supplementary Data 4

Reporting Summary

Transparent Peer Review file

## Data Availability

All transcriptomics datasets generated in this study have been deposited in the Gene Expression Omnibus (GEO) database. The total RNA high-throughput sequencing can be accessed under the accession number GSE313183. The small RNA sequencing can be accessed under the accession number GSE313185. The AGO2 IP sequencing datasets can be accessed under the accession number GSE313187. The transcriptomic datasets from FFPE-DICER1 tumours can be accessed at GSE328604. The raw proteomics datasets can be accessed at the Proteomics Identifications Database (PRIDE), under the accession number PXD072167. Additional datasets used in this study include miRNA and AGO2 iCLIP profiling of S1344L hESCs, GSE274853 and GSE274851, respectively. DICER structure was obtained from PDB ID 7XW2. microRNA expression in UCEC tumours was generated by data from TCGA Research Network [https://www.cancer.gov/tcga]. LINE-1 expression data were obtained from the TCGA pan-cancer analysis by ref. ^85^. Lists of studied genes, proteins and miRNAs are provided with this paper in the accompanying source and supplementary data.
