# Supplementary material for: DICER1-related tumour predisposition mutations increase 3p-miRNA function and HERVH activity
Source: Nat Commun. 2026 Jul 25;17:9045. doi: 10.1038/s41467-026-76094-2 (PMC13507251; doi:10.1038/s41467-026-76094-2)
Supplement: Supplementary file 1 [file 41467_2026_76094_MOESM1_ESM.pdf]

Supplementary Figure 1

a

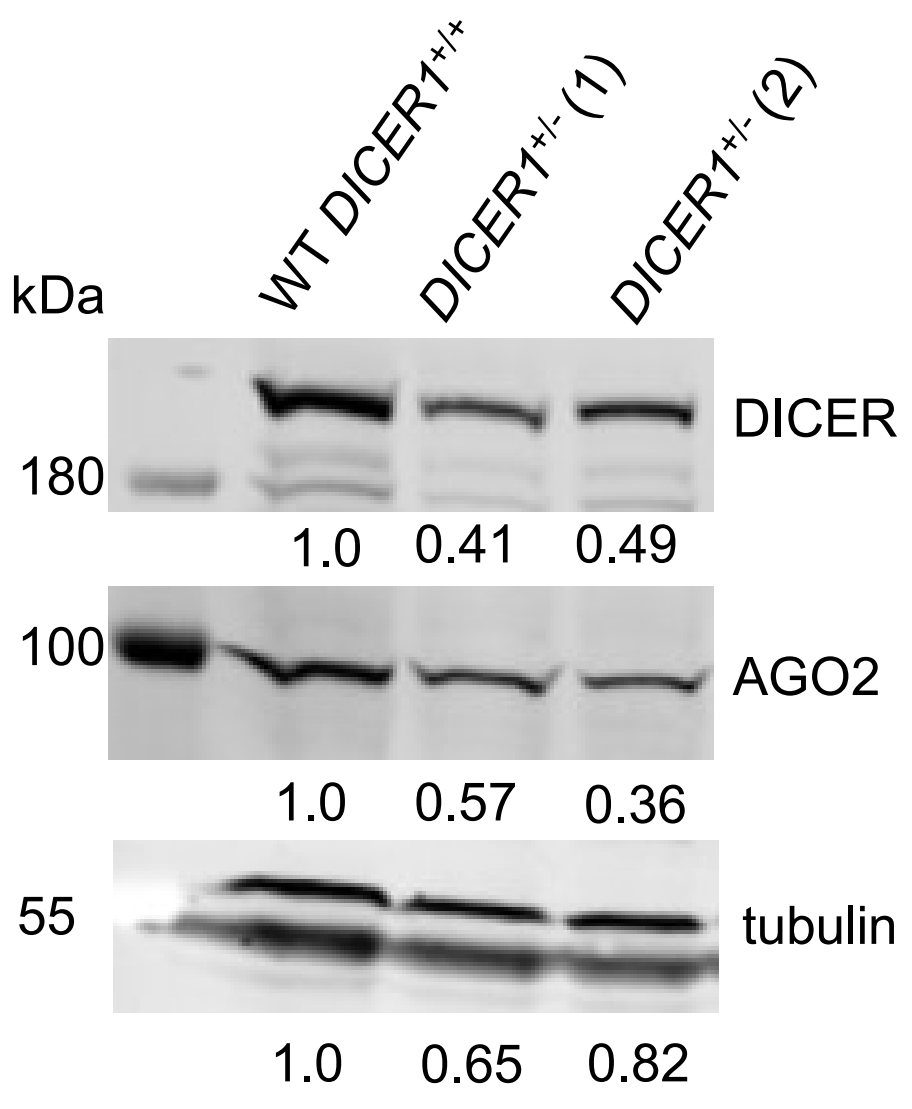

b

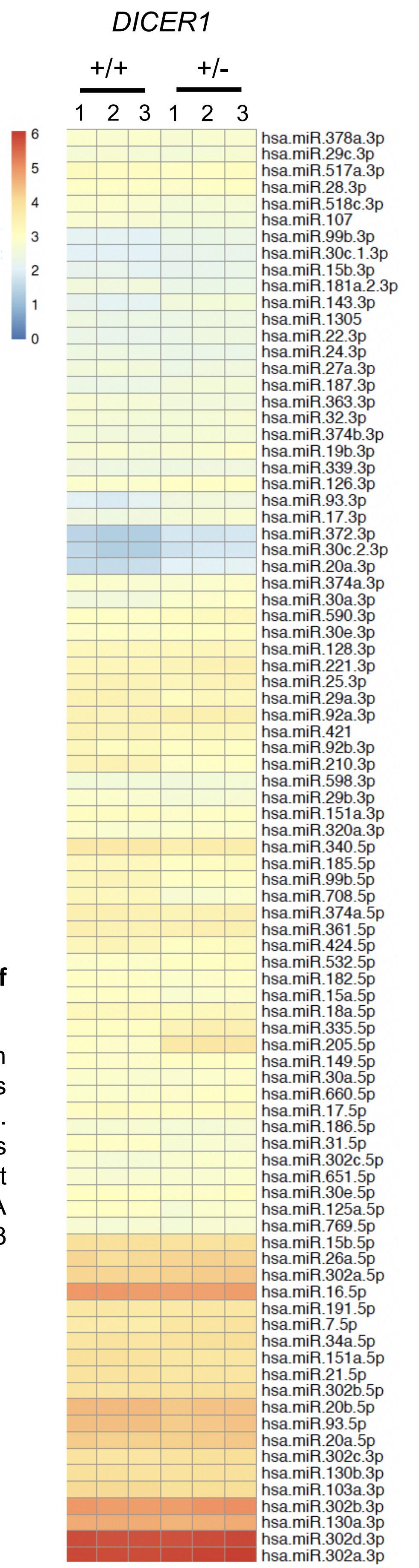

Supplementary Figure 1 Protein expression levels of DICER1 and AGO2 in DICER heterozygous cell lines

(**a**) Western blot analysis of DICER and AGO2 protein levels in WT (*DICER1*$^{+/+}$) and two independent *DICER1* Heterozygous (*DICER1*$^{+/-}$) clones with Tubulin as a loading control. Quantification vs WT cells is shown under each lane. Blots correspond to one representative experiment. Molecular weight (KDa) is indicated on the left (**b**) Heatmap for the miRNA expression of WT (*DICER1*$^{+/+}$) and *DICER1*$^{+/-}$ cells, containing 3 independent biological replicates each.

Supplementary Figure 2

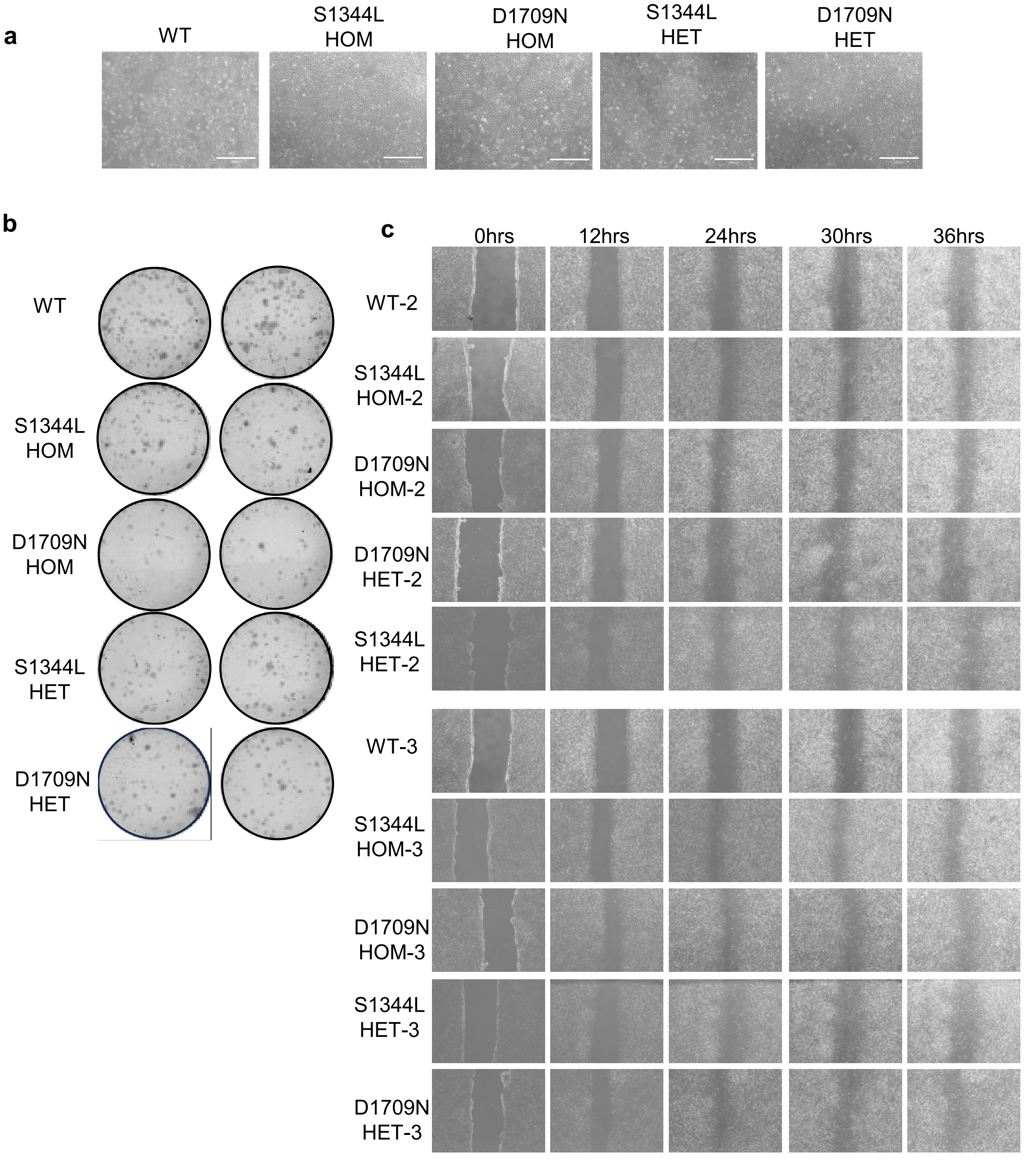

Supplementary Figure 2. Morphology, colony formation and wound healing capacity of *DICER1*-mutated cell lines (**a**) Morphology of the parental (WT) PA-1 cells and four of the different mutant cell lines generated. (**b**) Additional replicates of the colony formation assay shown in Figure 2a and b, making a total of n=3 biological replicates (**c**) Additional replicates of the wound healing time course shown in Figure 2e and f, making a total of n=3 biological replicates. Statistics information is included in main figure legend

Supplementary Figure 3

S1344L HOM

Gene.Name	Protein.ID	Log2FC	adj.P.Val	Significance	MIR-17-5P	MIR-93-5P
CCN1	O00622	1.11921811	0.00086844	+++ (P < 0.001)	+	+
CCN2	P29279	0.81496891	0.00055348	+++ (P < 0.001)	+	+
ITGAV	P06756	0.43286509	0.00023766	+++ (P < 0.001)	+	+
FSTL1	Q12841	0.68641989	0.00010309	+++ (P < 0.001)	+	+
DAB2	P98082	0.64936709	0.00585117	++ (P < 0.01)	+	+
SERPINE2	P07093	1.57515848	1.3149E-05	+++ (P < 0.001)	+	+
MMP14	P50281	0.71722095	0.00104288	++ (P < 0.01)	+	+
FERMT2	Q96AC1	0.32607859	0.00698887	++ (P < 0.01)	+	+
LRP1	Q07954	0.53539988	2.9326E-05	+++ (P < 0.001)	+	+

D1709N HOM

Gene.Name	Protein.ID	Log2FC	adj.P.Val	Significance	MIR-17-5P	MIR-93-5P
CCN1	O00622	0.7846219	0.00098798	+++ (P < 0.001)	+	+
CCN2	P29279	1.00457024	4.3487E-05	+++ (P < 0.001)	+	+
ITGAV	P06756	0.34918931	0.00043932	+++ (P < 0.001)	+	+
FSTL1	Q12841	0.6887819	6.4514E-05	+++ (P < 0.001)	+	+
DAB2	P98082	0.80703868	0.00147547	++ (P < 0.01)	+	+
SERPINE2	P07093	1.11141462	0.00019074	+++ (P < 0.001)	+	+
MMP14	P50281	0.57139394	0.00293945	++ (P < 0.01)	+	+
FERMT2	Q96AC1	0.2616312	0.00886609	++ (P < 0.01)	+	+
LRP1	Q07954	0.61614162	6.9056E-06	+++ (P < 0.001)	+	+

Supplementary Figure 3. Differentially expressed proteins associated with EMT and common to both mutant cells, S1344L and D1709N HOM. From left to right, the gene name, protein ID, log2FC changes in expression compared to WT cells, followed by adjusted p-value and significance are shown. Data are derived from four independent biological samples per cell line. Statistical analysis was performed by one-way ANOVA followed by Welch's t-test (adj p-val). Genes encoding these proteins contain predicted target sites for both miR-17-5p and miR-93-5p (as shown by '+')

Supplementary Figure 4

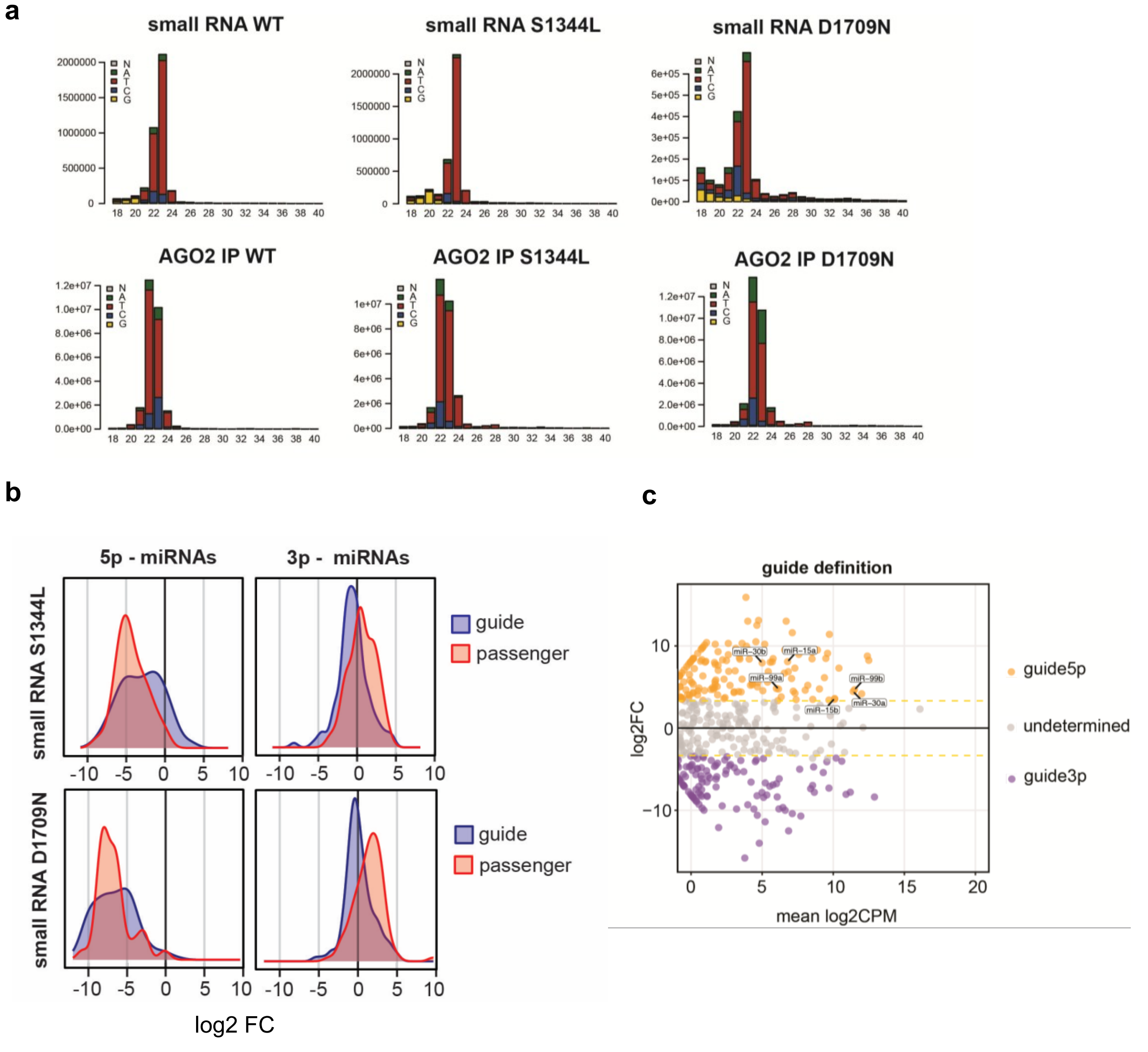

Supplementary Figure 4. miRNA expression analysis in the S1344L and D1709N mutants . (A) Read length distribution (18-40nt) of the small RNA high-throughput datasets (top) and AGO2 IP datasets (bottom) for WT, S1344L HOM and D1709N HOM cells. Only one representative library from one biological replicate is shown, but similar trend is observed with the additional replicates (total n=3). Colors indicate the identity of the first nucleotide. (**B**) Density plots for miRNAs (rows: miRNAs on 3p or 5p ends of hairpin, guide or passenger) in the small RNA dataset (WT, S1344L HOM, D1709N). Guide/passenger categories were defined based on expression in Ago2IP WT: if one arm had >10-fold more counts than the other, it was classed as guide and the other arm as star. Hairpins with arms with less than 10-fold differences were not classified. These data derives from at least n=3 biological replicates per cell line (**C**) MA plot displaying the log2 fold change (y-axis) of 5p and 3p miRNAs in WT PA-1 cells versus the average expression (*x*-axis, log2 counts per million, cpm). An arm is defined as a guide strand if it has 10-fold higher counts (indicated by yellow dashed line) than the other arm: 5p guide (yellow), 3p guide (purple), undefined (grey).

Supplementary Figure 5

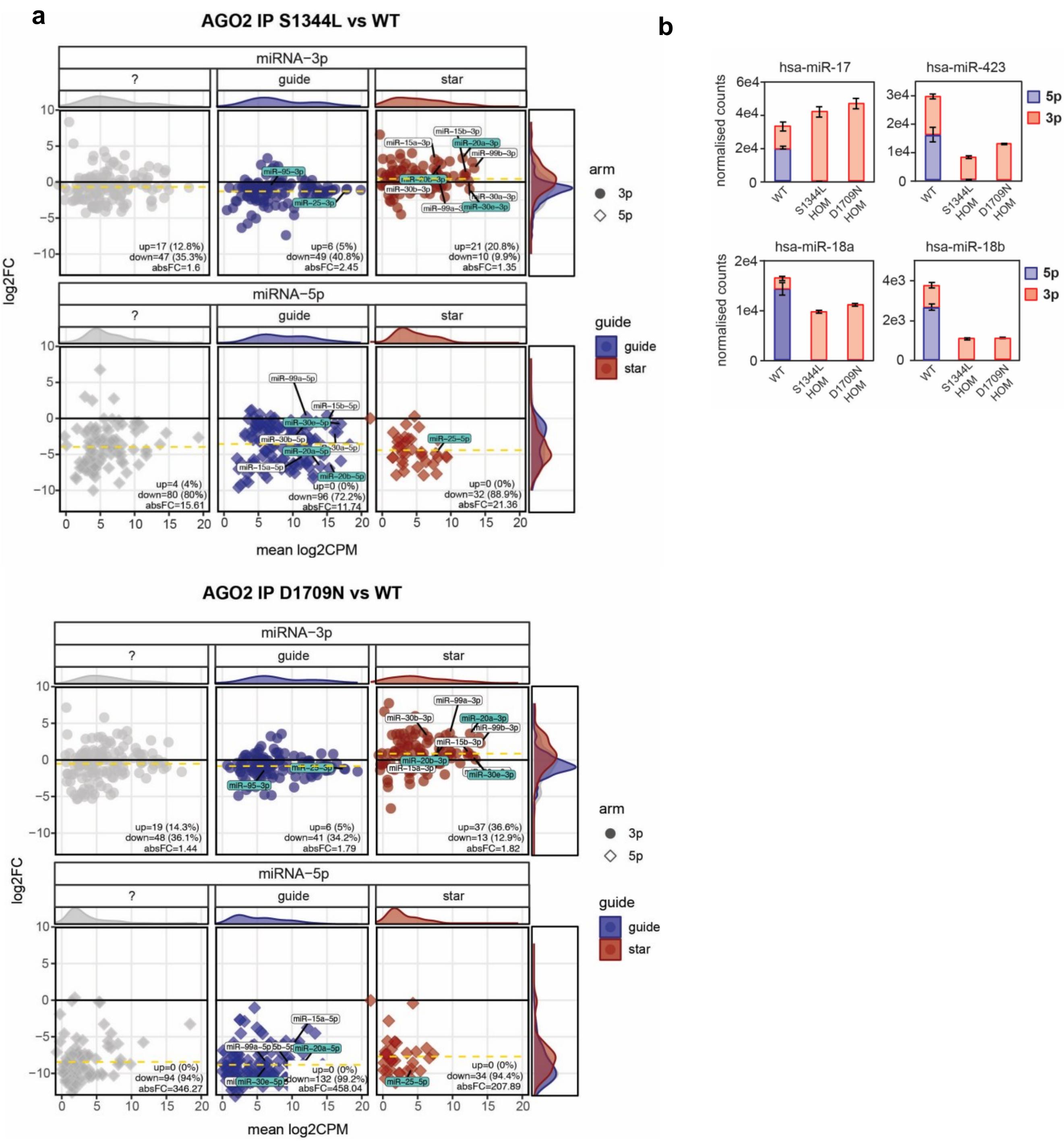

Supplementary Figure 5. Differential effect of D1709N and S1344L on guide and passenger 3p-miRNAs (**a**) Each panel represents the MAplot (*x*=log2CPM, *y*=log2FC) of a category of miRNAs (rows: miRNAs on 3p or 5p ends of hairpin, columns: ?, guide or star). Guide/star categories were defined based on expression in Ago2 IP WT: if one arm had >10-fold more counts than the other, it was classed as guide and the other arm as star. Hairpins with arms with less than 10-fold differences were not classified (? on the plot). Top and right projections show density plots for the panel or row, respectively. A selection of miRNAs are highlighted by name. Panel insets show the number (%) of significantly (FDR 5%) up and down-regulated miRNAs, and the absolute average fold-change for the category (absFC). **(B)** Examples of miRNA-AGO2 strand switching in *DICER1* mutant cells. AGO2-IP small RNA-seq data are represented as the average of normalized miRNA counts +/- SD. 5p miRNA in blue, and 3p miRNA in red. Average of normalized counts (N=3) +/- SD is shown.

Supplementary Figure 6

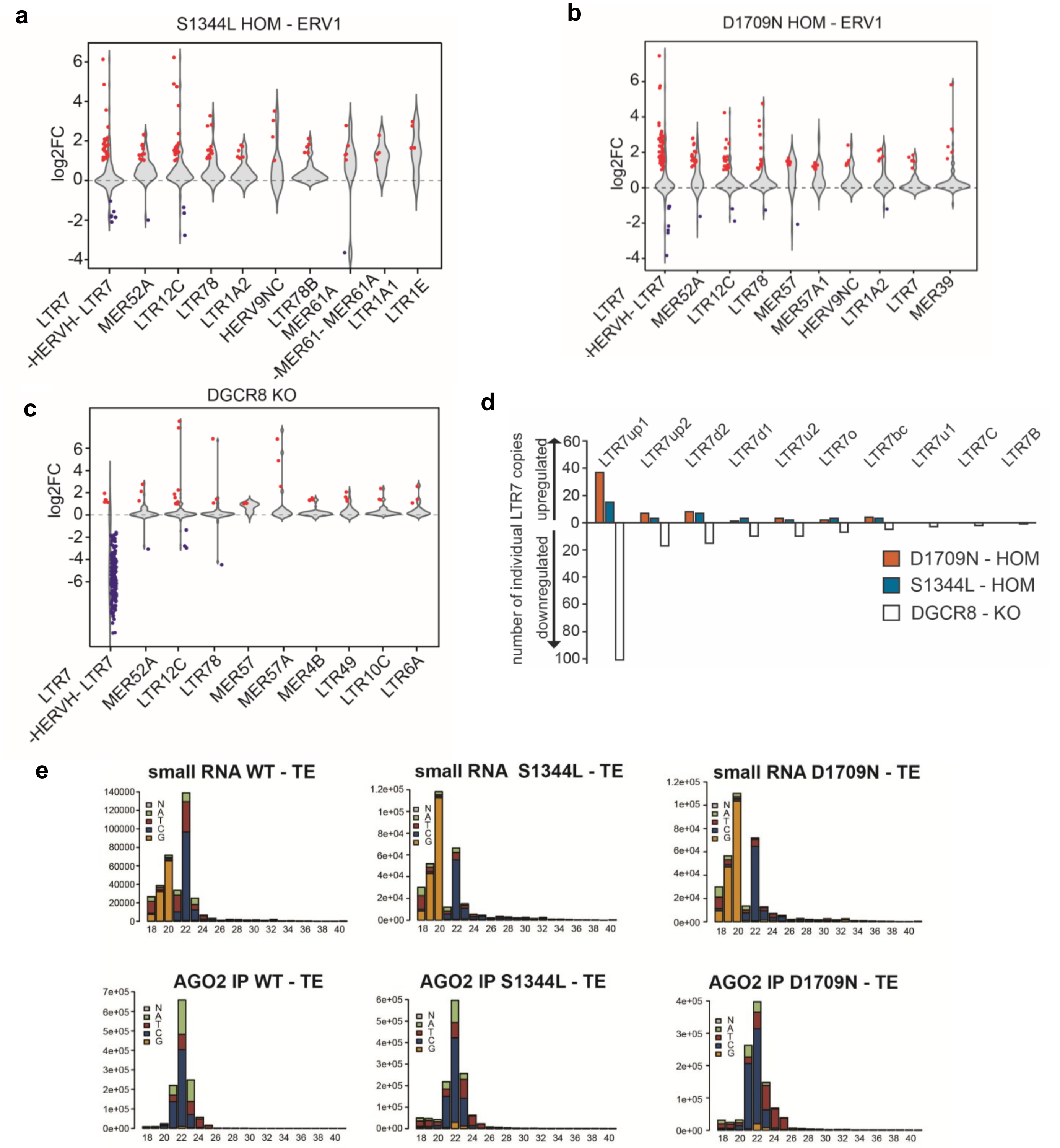

Supplementary Figure 6. ERV1 differential expression in *DICER1* and *DGCR8* mutant cells
(a) Violin plots for the differential expression analyses of the top 10 differentially expressed ERV in S1344L mutant, belonging to the ERV1 class. Red (upregulated) and blue (downregulated) dots represent copies that are significantly differentially expressed, by two-sided Wald test, p-adj <0.05 (**b-c**). The same as (**a**) for D1709N in (**b**) and *DGCR8* KO in (**c**). (**d**) Number of individual LTR7 copies belonging to each of the subfamily classification (LTR7up1 to LTRB) and that are significantly upregulated in both S1344L and D1709N *DICER1* mutants and downregulated in *DGCR8* KO cells, by two-sided Wald test, p-adj <0.05 (**e**) Average read sizes (10-40nt) of small RNAs mapping to TE in the small RNA high-throughput datasets (top) and AGO2 IP datasets (bottom) for WT, S1344L HOM and D1709N HOM cells. Only one representative library from one biological replicate is shown. Colors indicate the identity of the first nucleotide. Data with all biological replicates is included in Source Data file. All data derives from at least 3 biological replicates.

Supplementary Figure 7

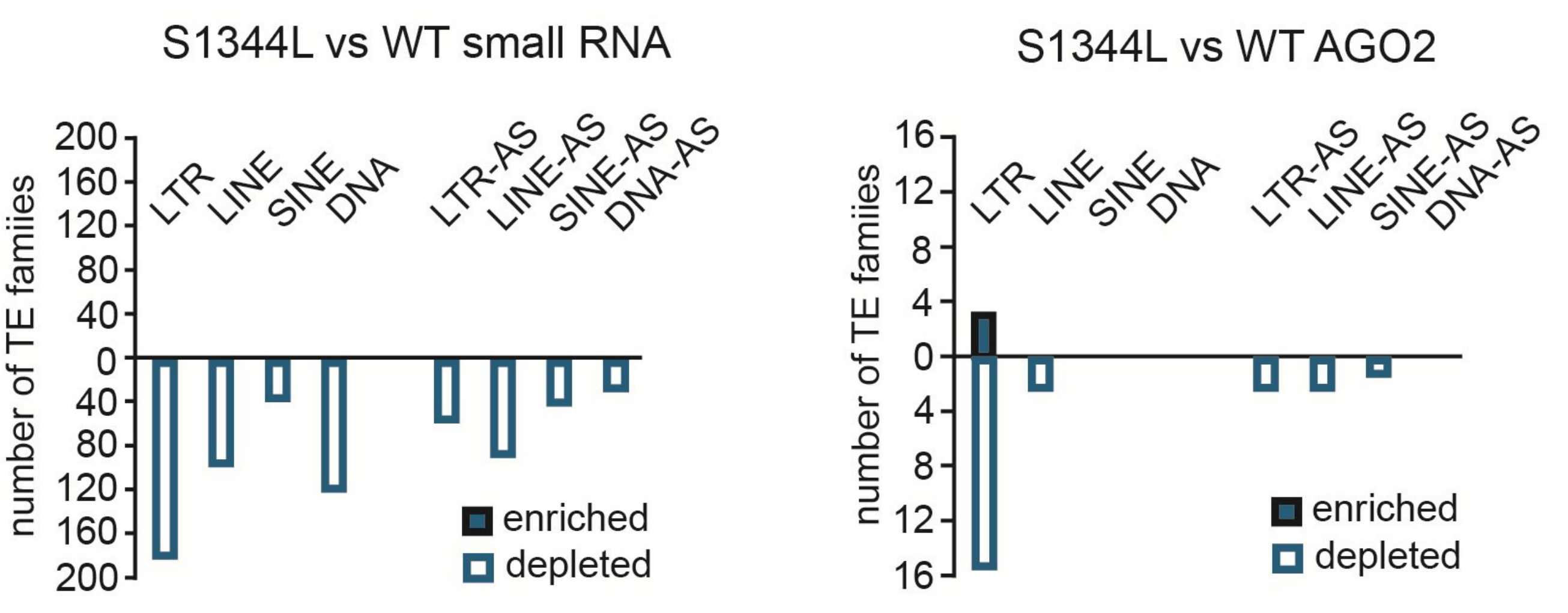

Supplementary Figure 7. Loss of TE-derived small RNAs in S1344L hESCs.
(left) Number of TE families that are differentially expressed in the small RNA high-throughput sequencing of S1344L homozygous mutant human embryonic stem cells (hESCs) versus WT cells (GSE274853 dataset from **1**). Families were classified in LTR, LINE, DNA and SINE classes. Upregulated (enriched) families are represented as positive counts, downregulated or depleted as negative counts. Reads mapping to all copies of each TE family were analysed separately by sense and antisense (AS) strands. (**right**) same analysis as in the left using the iCLIP AGO2 datasets of homozygous S1344L hESCs vs WT (GSE274851 dataset from **1**). Two biological replicates are used per cell line and experiment.

Supplementary Figure 8

healthy tissue	tumour tissue	DICER1 mutation
lymph node (S1N)	uterine embryonal rhabdomyosarcoma (S1T)	c.5113G>A p. (Glu1705K)
not available	ovary/Sertoli Leydig tumour (S2T)	c.615dupT p.(Thr206AsnsfTer10)
fallopian tube (S3N)	ovary/Sertoli Leydig tumour (S3T)	c.5113G>A p.(Glu1705K)

Supplementary Figure 8. Description of *DICER1*-mutated tumours and healthy tissues. Three *DICER1*-mutated tumours were obtained from Newcastle Biobank as formalin-fixed paraffin-embedded tissue (FFPE). Healthy tissue from two of the same patients was also obtained

Supplementary References

1. Jee, D. *et al.* Human DICER1 hotspot mutation induces both loss and gain of miRNA function. *Nat. Struct. Mol. Biol.* 1–11 (2025) doi:10.1038/s41594-025-01701-7.
