# Supplementary material for: DICER1-related tumour predisposition mutations increase 3p-miRNA function and HERVH activity
Source: Nat Commun. 2026 Jul 25;17:9045. doi: 10.1038/s41467-026-76094-2 (PMC13507251; doi:10.1038/s41467-026-76094-2)
Supplement: Supplementary file 2 [file 41467_2026_76094_MOESM2_ESM.pdf]

Description of Additional Supplementary Files

Supplementary Data 1. Total cell extracts proteomic (LC-MS/MS) analysis of S1344L HOM vs WT, and D1709N HOM vs WT. Differential protein abundance was calculated using 4 biological replicates for each cell line. Adj p-val was calculated using Benjamini-Hochberg False Discovery Rate (FDR) correction.

Supplementary Data 2. Total RNA high-throughput sequencing (RNA-seq) ribo-depleted analysis. Only common differentially expressed (DESeq2) between the comparison S1344L vs WT and D1709N vs WT mutants were included. UP stands for significantly upregulated in both mutants vs WT. DOWN are downregulated. At least n=3 biological replicates are used per cell line.

Supplementary Data 3. Small RNA high-throughput sequencing (small RNA-seq). Differentially expressed miRNAs for the S1344L vs WT and the D1709N vs WT comparisons are included. UP (upregulated) and DOWN (downregulated) miRNAs are separated in different tabs. At least n=3 biological replicates are used per cell line.

Supplementary Data 4. AGO2 immunoprecipitation datasets. AGO2 was immunoprecipitated in WT, S1344L and D1709N HOM mutants in triplicates (n=3 replicates). Associated RNA was analysed by high-throughput sequencing and differentially associated miRNAs for the S1344L vs WT and the D1709N vs WT comparisons are included. UP (upregulated) and DOWN (downregulated) miRNAs are separated in different tabs.
