# Supplementary material for: DICER1-related tumour predisposition mutations increase 3p-miRNA function and HERVH activity
Source: Nat Commun. 2026 Jul 25;17:9045. doi: 10.1038/s41467-026-76094-2 (PMC13507251; doi:10.1038/s41467-026-76094-2)
Supplement: Supplementary file 3 [file 41467_2026_76094_MOESM7_ESM.pdf]

nature portfolio

Corresponding author(s): Sara Macias

Last updated by author(s): Jul 13, 2026

Reporting Summary

Nature Portfolio wishes to improve the reproducibility of the work that we publish. This form provides structure for consistency and transparency in reporting. For further information on Nature Portfolio policies, see our Editorial Policies and the Editorial Policy Checklist.

Statistics

For all statistical analyses, confirm that the following items are present in the figure legend, table legend, main text, or Methods section.

n/a	Confirmed	
☐	☒	The exact sample size (*n*) for each experimental group/condition, given as a discrete number and unit of measurement
☐	☒	A statement on whether measurements were taken from distinct samples or whether the same sample was measured repeatedly
☐	☒	The statistical test(s) used AND whether they are one- or two-sided<br>*Only common tests should be described solely by name; describe more complex techniques in the Methods section.*
☒	☐	A description of all covariates tested
☐	☒	A description of any assumptions or corrections, such as tests of normality and adjustment for multiple comparisons
☐	☒	A full description of the statistical parameters including central tendency (e.g. means) or other basic estimates (e.g. regression coefficient) AND variation (e.g. standard deviation) or associated estimates of uncertainty (e.g. confidence intervals)
☐	☒	For null hypothesis testing, the test statistic (e.g. *F*, *t*, *r*) with confidence intervals, effect sizes, degrees of freedom and *P* value noted<br>*Give P values as exact values whenever suitable.*
☒	☐	For Bayesian analysis, information on the choice of priors and Markov chain Monte Carlo settings
☒	☐	For hierarchical and complex designs, identification of the appropriate level for tests and full reporting of outcomes
☒	☐	Estimates of effect sizes (e.g. Cohen's *d*, Pearson's *r*), indicating how they were calculated

Our web collection on statistics for biologists contains articles on many of the points above.

Software and code

Policy information about availability of computer code

Data collection	Image Studio Odyssey CLx<br>EVOS M7000 in-build image collection<br>Celigo image cytometer in-built image collection<br>Coot 0.9.6<br>TCGA biolinks
Data analysis	ImageJ v1.53q<br>Microsoft Excel<br>Prism 10.2.2<br>Celigo image cytometer in-build Colony application<br>EVOS 7000 in-build image acquisition software<br>Reaper<br>FeatureCounts<br>DESeq2, including lfcShrink function<br>STAR v2.7.9a and 2.7.11b<br>ShinyGO v0.81<br>Onecodetofindthemall.pl72<br>pullseq 1.02<br>Samtools 1.22.1<br>BEDtools 2.27.1<br>GDC Data Transfer Tool

Perseus v2.0.11
limma 3.62.2
Pymol 3.1.6.1
R v4.4.1

For manuscripts utilizing custom algorithms or software that are central to the research but not yet described in published literature, software must be made available to editors and reviewers. We strongly encourage code deposition in a community repository (e.g. GitHub). See the Nature Portfolio guidelines for submitting code & software for further information.

Data

Policy information about availability of data

All manuscripts must include a data availability statement. This statement should provide the following information, where applicable:

- Accession codes, unique identifiers, or web links for publicly available datasets
- A description of any restrictions on data availability
- For clinical datasets or third party data, please ensure that the statement adheres to our policy

All transcriptomics datasets generated in this study have been deposited in the Gene Expression Omnibus (GEO) database. The total RNA high-throughput sequencing can be accessed under the accession number GSE313183 (https://www.ncbi.nlm.nih.gov/geo/query/acc.cgi?acc=GSE313183). The small RNA sequencing can be accessed under the accession number GSE313185 (https://www.ncbi.nlm.nih.gov/geo/query/acc.cgi?acc=GSE313185). The AGO2 IP sequencing datasets can be accessed under the accession number GSE313187 (https://www.ncbi.nlm.nih.gov/geo/query/acc.cgi?acc=GSE313187). The transcriptomic datasets from FFPE-DICER1 tumours can be accessed at GSE328604 (https://www.ncbi.nlm.nih.gov/geo/query/acc.cgi?acc=GSE328604). The raw proteomics datasets can be accessed at the Proteomics Identifications Database (PRIDE), under the accession number PXD072167 (https://www.ebi.ac.uk/pride/archive/projects/PXD072167). Additional datasets used in this study include miRNA and AGO2 iCLIP profiling of S1344L hESCs, GSE274853 (https://www.ncbi.nlm.nih.gov/geo/query/acc.cgi?acc=GSE274853) and GSE274851 (https://www.ncbi.nlm.nih.gov/geo/query/acc.cgi?acc=GSE274851), respectively. DICER structure was obtained from PDB id 7XW2 (https://www.rcsb.org/structure/7XW2). microRNA expression in UCEC tumours was generated by data from TCGA Research Network (https://www.cancer.gov/tcga). LINE-1 expression data were obtained from the TCGA pan-cancer analysis by Solovyov et al. Lists of studied genes, proteins and miRNAs are provided with this paper in the accompanying source and supplementary data.

Research involving human participants, their data, or biological material

Policy information about studies with human participants or human data. See also policy information about sex, gender (identity/presentation), and sexual orientation and race, ethnicity and racism.

Reporting on sex and gender	All provided data is de-identified or pseudonymised and sex and gender were not relevant in this study
Reporting on race, ethnicity, or other socially relevant groupings	All provided data is de-identified or pseudonymised and no reporting of race/ethnicity or other relevant socially groupings was required for this study
Population characteristics	see above
Recruitment	Patient samples were selected based on identified DICER I mutations and compared with normal tissue from the same patient. Patient consent was requested prior to obtaining the samples
Ethics oversight	Archival FFPE tissue curls were obtained from Newcastle Hospitals Biobank. Generic ethical approval, was granted from the Newcastle and North Tyneside 1 Research Ethics Committee. Hospitals Biobank Access Committee assigned (Project Reference: CEPA182/CEPA223) for "Understanding the molecular consequences of Dicer mutations in Dicer I syndrome".

Note that full information on the approval of the study protocol must also be provided in the manuscript.

Field-specific reporting

Please select the one below that is the best fit for your research. If you are not sure, read the appropriate sections before making your selection.

☒ Life sciences ☐ Behavioural & social sciences ☐ Ecological, evolutionary & environmental sciences

For a reference copy of the document with all sections, see nature.com/documents/nr-reporting-summary-flat.pdf

Life sciences study design

All studies must disclose on these points even when the disclosure is negative.

Sample size	Sample sizes were based on variation observed in pilot experiments and the author's experience. For proteomics expert advice was sought on sample size. Statistical analysis of the experiments confirmed that the sample size was adequate.
Data exclusions	Data were only excluded in case of user error
Replication	At least three (n=3 or more) technical or (n=2 or more) biological replicates for all experiments were performed.

Randomization	Randomizations were not necessary for the type of experiments presented in this sutdy
Blinding	No blinding was necessary in this study as almost all data was quantitative and measured by machines. In cases where a certain level of subjectivity in observations might be present, the results were always verified by quantitative, machine derived data.

Reporting for specific materials, systems and methods

We require information from authors about some types of materials, experimental systems and methods used in many studies. Here, indicate whether each material, system or method listed is relevant to your study. If you are not sure if a list item applies to your research, read the appropriate section before selecting a response.

Materials & experimental systems

n/a	Involved in the study	
☐	☒	Antibodies
☐	☒	Eukaryotic cell lines
☒	☐	Palaeontology and archaeology
☒	☐	Animals and other organisms
☐	☒	Clinical data
☒	☐	Dual use research of concern
☒	☐	Plants

Methods

n/a	Involved in the study	
☒	☐	ChIP-seq
☒	☐	Flow cytometry
☒	☐	MRI-based neuroimaging

Antibodies

Antibodies used	Anti-tubulin (CP06, Millipore) 1:4000 (WB)<br>Anti-DICER (13D6, Abcam, AB13D6) 1:1000 (WB)<br>Anti-AGO2 (C34C6 Cell Signalling, cat 2897s) 1:1000 (WB)<br>anti-mouse 680RD (925-68070 LICORbio) 1:10000 (WB)<br>anti-mouse 800CW (926-32210 LICRObio) 1:10000 (WB)<br>anti-rabbit 680RD (925-68071 LICORbio) 1:10000 (WB)<br>anti-rabbit 800CW (926-32211 LICORbio) 1:10000 (WB)
Validation	all antibodies have been used and validated in other publications, please see company spec sheet for specific publications

Eukaryotic cell lines

Policy information about cell lines and Sex and Gender in Research

Cell line source(s)	PA-1 cells were originally purchased from ATCC CRL-1592
Authentication	Besides their distinctive epithelial like morphology, formation of embryoid like bodies, metaphase spreads were carried out showing a normal karyotype
Mycoplasma contamination	Regular testing were all negative
Commonly misidentified lines (See ICLAC register)	No commonly misidentified lines were used in this study

Clinical data

Policy information about clinical studies

All manuscripts should comply with the ICMJE guidelines for publication of clinical research and a completed CONSORT checklist must be included with all submissions.

Clinical trial registration	no data was collected
Study protocol	n/a
Data collection	n/a
Outcomes	n/a

Plants

Seed stocks	no plants were used
Novel plant genotypes	n/a
Authentication	n/a
