# Supplementary material for: DICER1-related tumour predisposition mutations increase 3p-miRNA function and HERVH activity
Source: Nat Commun. 2026 Jul 25;17:9045. doi: 10.1038/s41467-026-76094-2 (PMC13507251; doi:10.1038/s41467-026-76094-2)
Supplement: Supplementary file 4 [file 41467_2026_76094_MOESM8_ESM.pdf]

nature portfolio

Peer Review File

DICER1-related tumour predisposition mutations increase 3p-miRNA function and HERVH activity promoting metastatic potential

Corresponding Author: Dr Sara Macias

This file contains all reviewer reports in order by version, followed by all author rebuttals in order by version.

Version 0:

Reviewer comments:

Reviewer #1

(Remarks to the Author)
In this manuscript entitled "DICER1 syndrome mutations lead to a gain of 3p-miRNA function, HERVH activity and increased metastatic potential", the authors aim to understand the molecular mechanism underlying the inherited cancer-predisposing disorder called Dicer1 syndrome. Individuals with this syndrome have an increased risk of developing tumors and patients typically carry a germline loss-of-function mutations in one allele and then acquire a somatic mutation in hotspots including changes in the RNAse III a or RNAse IIIb catalytic domains. To study the role of Dicer1 syndrome-associated mutations, the authors used a CRISPR/Cas9 genome editing approach to generate human embryonic cell lines (PA-1 cells) that were either homozygote (HOM) mutants (i.e. harboring a single substitution in the RNAse IIIa domain (S1344L) or in the RNase IIIb domain (D1709N) in both Dicer 1 alleles) or heterozygote (HET) mutants (i.e.S1344L or D1709N mutation in one allele ,while the second allele contains a loss-of-function mutation). They found that, while mutant cells had a lower replication rates and a decreased ability for colony formation, they had increased metabolic activity and ability to migrate using a wound healing essay. They then analysed the common dysregulated genes in Dicer1 mutant cells and identified an enrichment for pathways that support an increased in migration potential (i.e epithelial to mesenchymal transition, KRAS signalling). Since Dicer is an essential enzyme for the biogenesis of miRNAs, the authors then analysed the impact of Dicer mutations on miRNAs. They found that while both mutations caused a decreased level of 5p-miRNAs, it resulted in higher abundance of some 3p-miRNAs by both total small RNA deep sequencing and small RNA-seq of Argonaute 2 (AGO2) immunoprecipitation. These changes in 5p-miRNAs and 3p-miRNAs were then shown to have a functional impact on their predicted target mRNAs. Finally, the authors found that in both Dicer1 mutant cell lines, human endogenous retrovirus H (HERVH) transposable elements were upregulated through a miRNA-independent mechanism.

The findings from Gordon et al. are interesting and provide important insights into our understanding of how hotspot mutations found in Dicer1 syndrome predispose to cancer. While the experiments are overall well-conducted and the data support the authors' conclusions, some additional experiments should be performed to strengthen their findings. Additionally, greater clarity is needed in parts of the manuscript.

Here are specific comments:

• Figure 1c, 1d and 1f: the authors mentioned that there is no significant differences of Dicer levels between PA-1 cells HET and HOM mutants. While the data indeed tend to show that there are no significant differences, statistical tests should be included in Figure 1d to fully assess the significance of any variations seen in Dicer levels. There seem to be a decreased level of Dicer in both HOM and HET cell lines compared to WT cells. While there are no statistical tests in figure 1d, the proteomic data also show that both HOM cells lines have a reduced level of Dicer compared to WT and that this decreased level is statistically significant. The authors should clarify in their manuscript that there are no differences in Dicer levels between HET and HOM but overall the level of Dicer is reduced compared to WT.

• Figure 2G and 2H: It is unclear which cell lines are used for the RNA-seq analyses. In the figure legend, it is mentioned that genes in "Dicer1 mutants" cells are compared to WT PA-1 cells. In the manuscript, it is written that RNA-seq analyses were carry out in both S1344L and D1709N cell lines compared to WT (lines 145-146). Can the authors clarify whether the HET or HOM Dicer1 cells were used for RNA-seq analyses? The HET cells are more representative of the genetic alterations

occurring within tumors of Dicer1 syndrome so it would be preferable to analyse HET mutant cells. However, this reviewer appreciates that the level of Dicer expression was equivalent between both HOM and HET cells (figure 1) and therefore S1344L/ D1709N HOM or HET cells should express similar levels of Dicer mutant proteins anyway. In addition to mentioning in the manuscript and in the figure legends the exact Dicer1 mutant cells used, the authors should explain briefly within the manuscript their rationale for either HOM or HET Dicer-1 mutant cells. Same comment for Figure 3a and 3b, please clarify which Dicer1 mutant cells were used (i.e. S1344L HOM or HET, D1709N HOM or HET) for these analyses.

• Figure 3: The observations that in both Dicer1 mutant cells (S1344L or D1709N), there is an overall decrease of 5p-miRNA strands (guide and passenger) and an increase of 3p-miRNA passenger strand are interesting and constitute a key finding of the manuscript. It would strengthen the authors' findings to perform small Northern blot or qRT-PCR analyses on specific miRNA strands. While the authors nicely show in figure 3e the abundance of four specific miRNAs including the abundance of their respective 5p and 3p strands, the data is derived from the same RNA-seq analysis shown in Figure 3a. It would reinforce the findings to confirm these changes in strands abundance using well-established qRT-PCR or small RNA Northern blotting approaches on Dicer1 mutant cells compared to WT.

• Figure 3a,b: the authors show that while that 3p-miRNAs guide strands are not altered in Dicer-1 mutant cell lines, the 3p-miRNA passenger strands are upregulated. Are all 3p-miRNAs passenger strands increased in their abundance? If not, which proportion is upregulated? Could the authors compared the sequence of 3p-miRNAs passenger strand that get upregulated compared to those whose level are unchanged or downregulated to determine if there is an enrichment for sequences containing a specific 5'end or other features?

• Figure 3b: The authors should include a western blot to show that AGO2 is efficiently and specifically immunoprecipitated in WT as well as in Dicer1 mutant cell lines and that Ago2 is immunoprecipitated at a similar level between cell lines (mutants vs WT).

• Figure 4a and 4b: The authors assessed whether the accumulation of 3p-miRNAs resulted in increased RNA silencing of their target mRNAs. For this, they carried out a cumulative distribution functon(CDF) analysis on a set of 3p and 5p-miRNAs predicted target mRNA. The authors should clarify what the black distribution represents, i.e. what is the control group in their analyses: Did they use, as a control, untargeted genes, i.e. genes that are neither target of upregulated 3p-miRNA nor predicted targets of downregulated 5-miRNAs? If so, how many untargeted genes were included in the control? Please include these information within the figure legend. Additionally, it is currently stating, within the figure legend, that the data shows the CDF vs fold changes of predicted gene targets for upregulated (red) and downregulated (blue) miRNAs in Dicer-1 mutant cells. Does it indeed show the change in the predicted target of all upregulated or downregulated miRNAs from Dicer-1 mutant cells? I assume that in red it shows the specific distribution of predicted targets from specifically 3p miRNAs (not all miRNAs) that are found to be upregulated in Dicer1 mutant cells, whereas, in blue, it corresponds to predicted targets of downregulated 5p miRNAs. Please clarify the figure legends accordingly.

• Figure 5d-f: the authors detected small RNAs derived from various families of transposable elements (TEs) loaded into AGO2. Dicer generates small RNAs of 21-23 nucleotides in length, what are the length of the small RNAs isolated from AGO2? What are the number and length of these various TE-specific small RNAs before IP (i.e. in RNA extracts)? Are the identified TE-specific small RNAs of 22-nt in length depleted in Dicer-1 mutant cells?

• The authors found that the identified TE-specific small RNAs are mainly depleted from AGO2 IP carried out in Dicer1 S1344Lor D1709N mutant cells versus Ago2 IP from wild-type cells. However, the reads derived from tRNAs are also depleted in the S1344L AGO2 IP versus WT cells (figure 3e). While some tRNAs were previously shown to be processed by Dicer into tRNA-derived small RNAs, can the authors rule out that AGO2 proteins were less efficiently immunoprecipitated in Dicer-1 mutant cells compared to WT cells?

• Figure 5g: the authors found that in Dicer1 mutant cell lines there is a change in the composition and level of AGO2-associated small RNA derived from LTR7up2 subfamily of HERVH. While there is a modest decrease in the abundance of the antisense and sense RNA reads in the 3' end of LTR7up2, there is an increase abundance of other antisense small RNAs mapping at another upstream region. If these antisense small RNA were Dicer-dependent, we would anticipate to have a depletion of TE-derived small RNAs in AGO2 IP. Are these increased antisense reads of 21-23 nucleotides (i.e. potentially Dicer-dependent)in length or are their lengths different (i.e. potentially Dicer-independent)?

Minor comments:

• Supplementary Figure 1a: Please add the name of the proteins (i.e. Dicer, Ago2 and Tubulin) being detected on the right-hand side of the western blot.

• Lines 141-142: the authors should briefly explain how the metabolic activity is measured in the Dicer1 mutant cell lines, i.e. by using a luminescence-based assay to measure the level of intracellular ATP.

• Figure 3e: it is unclear if the data represent the abundance of miRNA by total small RNA-seq or the amount of miRNA loaded into AGO2 by RNA-seq analysis on AGO2-IP. Please clarify in the figure legend.

• Figure 4e,f and 3g,h: please add in the figure legend the statistical test used in your approach and that the Benjamini-Hochberg False Discovery Rate (FDR) value is depicted in the figure.

• Figure 5g: Please add a legend (small RNA counts) and scale on the y-axis.

Reviewer #2

(Remarks to the Author)

The authors generated human cell lines containing two different “hotspot” missense mutations in DICER1, a miRNA processing gene. They showed loss of 5p and gain of 3p passenger (3p*) strands for both mutations, altering gene expression profiles to favour tumorigenesis. The comparison of S1344L and D1709N is novel and allows for some new and potentially important insights. They also report the novel finding that these mutations cause upregulation of HERVH likely through a non-miRNA-related mechanism.
This is an interesting paper with some novel findings discussed above that I enjoyed reading.
Some comments, thoughts and suggestions
Major points
1) PA-1 is diploid, but it is transformed and immortalized, derived from a teratocarcinoma cell line and can tumors in nude mice. According to various sources, they are highly proliferative. To use a carcinoma cell line as a model system for human DICER1 seems risky. I suggest that the authors validate all their findings using a cell line such as IMR90 that is diploid and is a fibroblast line, thus much more likely to be relevant for DICER1-related tumors, which are mainly mesenchymal in origin. This is also very important for the data showing de-repression of TEs. Does this occur in non-transformed cells?
2) Line 360 et seq. The authors raise an important point. None of this work has been confirmed in human DICER1-related tumors. It would enormously strengthen this work if this work could be done for more conventional parts of the manuscript and especially for the newer TE-related findings.
Other important points
3) Design of the study:
Comparing S1344L and D1709N mutations was an inspired decision. But I cannot see in this paper the rationale for this choice. What was the thinking behind this – what was hoped to be revealed? Is S1344L relevant in humans?
Results:
4) the results are presented as first mRNA changes, then miRNA changes. It would be easier to follow the work if the miRNA data were presented first, then the mRNAs, with overlaps between S1344L and D1709N described first for miRNA then mRNA, so that the path from the mutation to the downstream effect is more clearly delineated. Then the cellular work can be the final section for this part of the work.
5) Direct evidence that the a) upregulated and b) downregulated transcripts are in fact driving the observed behaviour of these cells is lacking. For a), EMT and KRAS signalling is said to “be associated with” and “to agree” with observed effects on wound healing, and “may explain” enrichment of the myogenesis pathway, but a direct link is not provided. That the mRNAs are implicated in EMT and KRAS signalling is one thing, but experimental validation is lacking. For b) it is a similar story.
6) Lines 175-190. It is not clear from the way the text is written which parts of this section are new results specific to this paper, and which results have been presented by Vedanayagam, J. et al. in 2019, by V. Narry Kim in Nature (2023) and more recently by the Lai lab in Nat Struct Mol Biol. All these sources are appropriately cited, it is just not completely clear which parts are new to this paper.
7) Lines 191-212. While it is clearer here what is new to this paper, the results are confirmatory of the two recently published papers that are cited. This undermines the opening sentence, as the results cannot be considered to be “unexpected”. I would suggest this section be rewritten as new data, without editorializing, and more the references to the discussion, acknowledging that you are confirming published data.
8) Lines 213- 223. The more important question to my mind is whether these AGO2-loaded 3p passenger strands are actually doing anything. The issue here is that Figure 4A and B are based on predicted targets. How reliable is this, especially for 3p passenger strands? And how do you know the effects are via “destabilization” rather than some down-stream effect of altering 5p or 3p levels? This is relevant as there appears to be an effect of loss of 5ps on the proteome for S1344L but not on the transcriptome. Mechanistically, how would this be happening?
9) Lines 224-231. Some experimental validation of these predicted targets would strengthen the case for EMT (5p) and metabolic (3p) derangement.
10) Lines 232 et seq. This work is by far the most novel data presented. The difference in effect for DGCR8 KO and these mutants is intriguing and makes me think that is might be important to repeat the experiments with DICER1 KO cells and with DICER1 D1320A or E1564A mutated cells to see if this is related to the hotspots per se (I realise PA-1 KO cells were not obtained, but see the first point above). Or repeat using murine Dicer1 KO cells. Add-back experiments to see if the effect can be reversed by competition with WT DICER1 could be valuable. The authors conclude “All these together suggest that mutations of the endonucleolytic function of DICER1 alter loading of sense and antisense small RNAs that are complementary to transcripts derived from TEs, regulating their post-transcriptional gene silencing”. This is a fascinating conclusion, the significance of which is hampered the lack of data to show that TE de-repression happens in human DICER1-related tumors, and if this is the case, what is the significance of this finding (see point 2 above).
Minor points
Introduction:
11) “Considering that DICER1 is an essential protein for cell survival, the observation that inactivating its catalytical activity can contribute to cancer is counterintuitive25,26”
- Given the function of the catalytic sites in the IIIB domain is to cleave pre-miRNAs, it would seem that inactivating this

function would distort the miRNA environment, with unpredictable consequences. So, I wondered why the authors thought this was counter-intuitive – does this come from refs 25 and 26?
12) "Mechanistically, we show that DICER1-associated mutations do not cause genome instability or chromosome abnormalities, as suggested for Dicer knockout cells". Also lines 235-235 (results, with citations).
Ok. But this is not expected in human tumors. See Pugh et al, Oncogene or the work of David Huntsman. Genome instability is not a typical feature of human DICER1-related tumors, so this needs to be acknowledged. See point 2 as well.
Results:
13) "DICER1+/- PA-1 cells, where only a single allele is inactivated, do not display significant changes in miRNA expression, arguing against DICER1 haploinsufficiency". The original mouse work is cited, but please comment on this finding in the context of the more recent paper of Mark Hatley's group (Dev Cell, 2026).
14) Nomenclature:
DICER1 syndrome is now properly referred to as "DICER1-related tumor predisposition".
(https://monarchinitiative.org/MONDO:0100216).

Reviewer #3

(Remarks to the Author)
Gordon and colleagues present nice work on the effect of different DICER1 mutations on miRNA processing, transcriptome regulation and retrovirus regulation. The experiments and the design of the manuscript is a credit to the authors, and the majority of the work is clear, well-presented and with reasonable controls. Their findings that 3p miRNA sequences displace 5p sequences in these mutants was particularly interesting. Despite the clear quality of this work, some minor refinements would improve readability and its impact.
1. Figure 3 provides nice data; however, its description and the order the data is discussed in the results is hard to follow. Information on the guide and passenger strand is not presented when the figure is described. This stalls the flow of paper and needs a re-think.
2. In Figure 4E/F, is this data solely from D1709? As S1344L doesn't change transcriptome or proteome but does have 3p miRNA switching this clarification and some description should be added to the text. Clearly, D1709 affects the transcriptome and retroviruses, but S1344L only affects retrovirus. This discrepancy and how S1344L affects cellular phenotypes (Figure 2) that appear independent of retrovirus function is a significant gap.
3. Based on these differences, between the mutations, clarifying statements on how they affect cells and the retrovirus activation would be useful.
Minor 1: Figure 2G/H need clarifying labels
Minor 2: Stats are missing from Figure 2A.
Overall, this is an exciting manuscript that requires a couple of refinements to increase its accuracy and contribution to the field.

Version 1:

Reviewer comments:

Reviewer #1

(Remarks to the Author)
I thank the authors for their rebuttal. The authors have satisfactorily addressed all my previous concerns and I therefore recommend acceptance of the manuscript. I note one minor typo in line 1032, where "5p/30" should be changed to "5p/3p".

Reviewer #2

(Remarks to the Author)
Impressive revisions!

I have some new questions which mainly relate to the additional data provided.

line 320: To this end, we first compared 5p and 3p miRNA expression in TCGA tumor datasets with homozygous DICER1 mutations..

My question: which tumors have homozygous mutations? Do you mean homozygous or hemizygous? It would be good to have more details. As you know, not all DICER1 mutations are equal! See comments below.

line 324: three different tumours containing DICER1 mutations, two with mutations in the RNAse IIIb (E1705K) and one with

a frameshift mutation resulting in a truncated protein lacking both RNAse III motifs (Figure 6B and Suppl. Fig 8).

my question: tumor S2T appears to have one LOF hit. Like the normal cells in a person with DRTP. How does his lead to deranged miRNAs?

line 331: the two active LINE-1 elements in humans, was increased in HNSC (head and neck squamous cell carcinoma) and LUAD (lung adenocarcinoma) samples from the TCGA repository. This effect was observed when comparing tumours containing DICER1 mutations versus non-mutated tumours (Figure 6E).

my question: HNSC and LUAD are not commonly (if ever) reported in DRTP (except for fetal types) - can you list the actual DICER1 variants seen in these 53 tumors? I just want to know what you mean by DICER1 mutated. Is having a IIIB or IIIA hotspot enough? or do they have to have to 2 hit to generate the TE effect?

Additional question: surely other tumors in TCGA must have DICER1 mutations like the ones you used in the analysis here. Can you explain why you chose these tumors for further analysis?

line 703: differential expression analysis of DICER1 mutated tumour samples (n=14) versus non-DICER1 mutated...

my question: is this number (14) lower than the 53 above because miRNA data is only available for a small number of tumors in TCGA?

Figure 4g-j - can you explain why S1344L homozygous behaves differently to S1344L heterozygous in some of the reporter assays? And are those differences significantly different? They look to be so for h, i and j.

Another question that comes to mind regarding the TE part of the ms (that I should have thought of before in fact) - given the role of piRNAs in TE regulation, can you compare the piRNA-TE world with that of the DICER1-TE world that you are describing? It seems worthy of a discussion point.

----

minor comments: lines 24, 77, 356: the syndrome is not called DICER1-related tumo(u)r predisposition (DTRP) syndrome, but just DRTP. this is for complicated reasons, but please just use DRTP without "syndrome". You might want to use the abbreviation.

Reviewer #3

(Remarks to the Author)
The authors have done a nice job of responding to my comments and I feel that the paper reads well.

Open Access This Peer Review File is licensed under a Creative Commons Attribution 4.0 International License, which

permits use, sharing, adaptation, distribution and reproduction in any medium or format, as long as you give appropriate credit to the original author(s) and the source, provide a link to the Creative Commons license, and indicate if changes were made.
In cases where reviewers are anonymous, credit should be given to 'Anonymous Referee' and the source.
The images or other third party material in this Peer Review File are included in the article's Creative Commons license, unless indicated otherwise in a credit line to the material. If material is not included in the article's Creative Commons license and your intended use is not permitted by statutory regulation or exceeds the permitted use, you will need to obtain permission directly from the copyright holder.
To view a copy of this license, visit https://creativecommons.org/licenses/by/4.0/
